# Type II kinase inhibitors that target Parkinson’s disease–associated LRRK2

**DOI:** 10.1126/sciadv.adt2050

**Published:** 2025-06-04

**Authors:** Nicolai D. Raig, Katherine J. Surridge, Marta Sanz-Murillo, Verena Dederer, Andreas Krämer, Martin P. Schwalm, Nicholas M. Lattal, Lewis Elson, Deep Chatterjee, Sebastian Mathea, Thomas Hanke, Andres E. Leschziner, Samara L. Reck-Peterson, Stefan Knapp

**Affiliations:** ^1^ Institute of Pharmaceutical Chemistry, Goethe University, Max-von-Laue-Str. 9, 60438 Frankfurt am Main, Germany.; ^2^ Structural Genomics Consortium (SGC), Buchmann Institute for Life Sciences, Max-von-Laue-Str. 15, 60438 Frankfurt am Main, Germany.; ^3^ Aligning Science Across Parkinson’s (ASAP) Collaborative Research Network, Chevy Chase, MD 20815, USA.; ^4^ Department of Cell and Developmental Biology, School of Biological Sciences, University of California San Diego, La Jolla, CA 92093, USA.; ^5^ Department of Cellular and Molecular Medicine, School of Medicine, University of California San Diego, La Jolla, CA 92093, USA.; ^6^ Department of Molecular Biology, School of Biological Sciences, University of California San Diego, La Jolla, CA 92093, USA.; ^7^ Howard Hughes Medical Institute, Chevy Chase, MD 20815, USA.

## Abstract

Increased kinase activity of leucine-rich repeat kinase 2 (LRRK2) is associated with Parkinson’s disease (PD). Numerous LRRK2-selective type I kinase inhibitors have been developed, and some have entered clinical trials. Here, to our knowledge, we present the first type II kinase inhibitors that target LRRK2. Targeting the inactive conformation of LRRK2 is functionally distinct from targeting the active-like conformation using type I inhibitors. We designed these inhibitors with a combinatorial chemistry approach fusing selective LRRK2 type I and promiscuous type II inhibitors using iterative cycles of synthesis supported by structural biology and activity testing. Our lead compounds are selective and potent toward both LRRK2 and LRRK1, a close relative of LRRK2. Through cellular assays, cryo–electron microscopy structural analysis, and in vitro motility assays, we show that our inhibitors stabilize the open, inactive LRRK2 kinase conformation. These new conformation-specific compounds will be invaluable as tools to study LRRK2’s function and regulation and expand the potential therapeutic options for PD.

## Teaser

LRRK2 type II kinase inhibitors stabilize the inactive kinase form, offering tools to study and treat Parkinson’s disease.

## INTRODUCTION

Parkinson’s disease (PD) is a progressive neurodegenerative disorder affecting more than 6 million people globally (*1*, *2*). While most PD cases are idiopathic with no known cause, more than 5% are familial, linked to specific gene mutations (*3*, *4*). Mutations in leucine-rich repeat kinase 2 (LRRK2) are a common cause of familial PD (*5*–*8*). LRRK2 kinase hyperactivity has also been described in idiopathic PD cases (*9*), making LRRK2 a key target for PD research and therapeutic intervention.

**Fig. 1.**
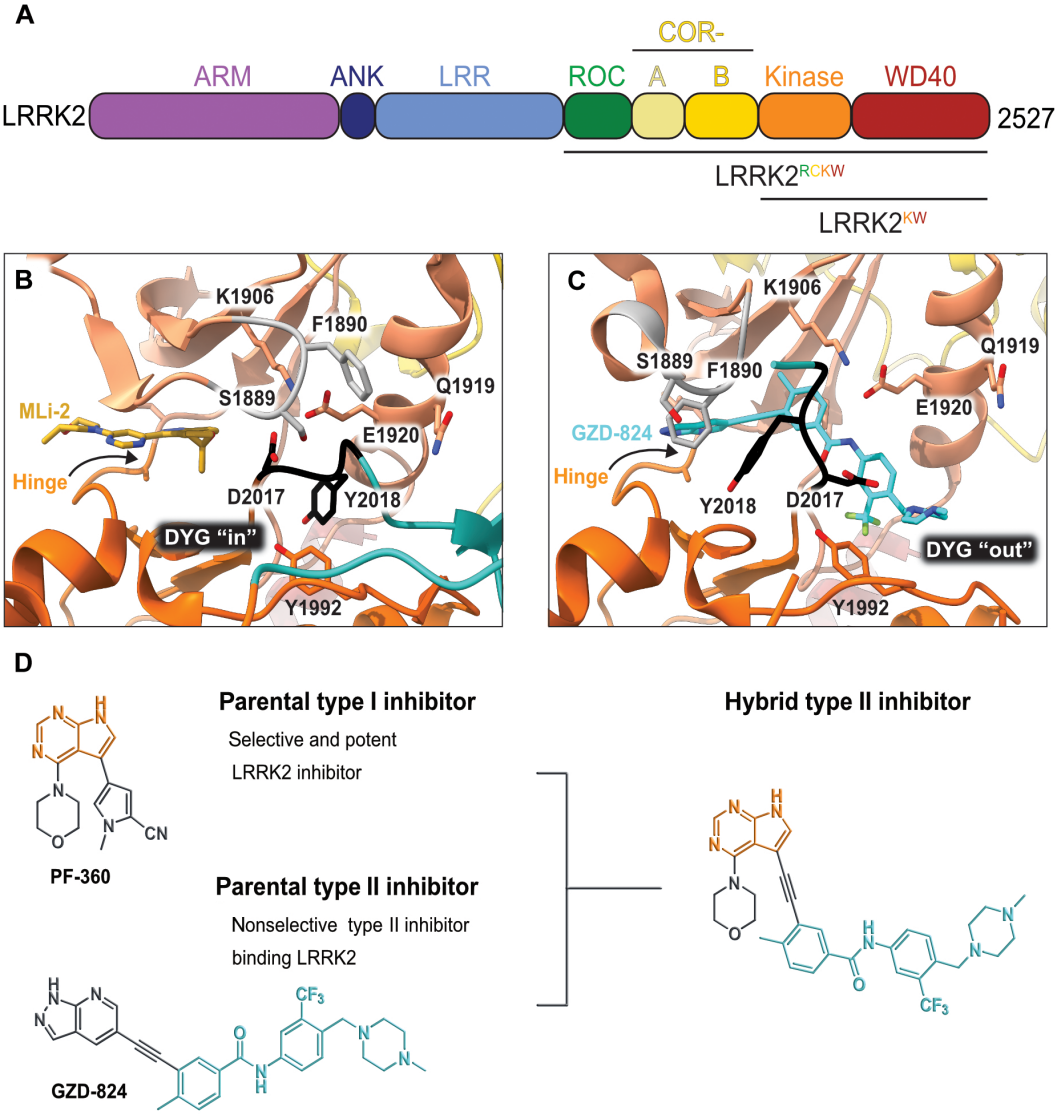
LRRK2 type II inhibitor design strategy. (**A**) Schematic domain structure of LRRK2. The three constructs used in this study are indicated: full-length LRRK2, LRRK2^RCKW^, and LRRK2^KW^. (**B** and **C**) Close-up of the inhibitor binding pocket from cryo–electron microscopy (cryo-EM) maps and models of LRRK2^RCKW^ bound to the type I inhibitor MLi-2 [Protein Data Bank (PDB): 8TXZ] (B) and type II inhibitor GZD-824 (PDB: 8TZE) (C). Key residues and features are labelled. Both structures are shown in the same view, aligned through the C-lobe of the kinase. Dark orange, C-lobe; light orange, N-lobe; black, DYG motif; gray, G-loop; green, activation loop. (**D**) Scheme depicting our hybrid design strategy to develop potent type II inhibitors targeting LRRK2.

LRRK2 is a large, multidomain protein. At its N terminus, LRRK2 contains armadillo (ARM), ankyrin (ANK), and leucine-rich repeat (LRR) domains. Its catalytic, C-terminal portion is made up of Ras of complex (ROC), C-terminal of ROC (COR), kinase, and WD40 domains (Fig. 1A). Most pathogenic mutations in LRRK2 are located within the catalytic portion, termed LRRK2^RCKW^ for **R**OC, **C**OR, **K**inase, and **W**D40 (acronym letters bolded). Of these, the G2019S mutation is the most common variant associated with PD. This mutation is located in the kinase active site and leads to an aberrant increase in kinase function (*10*–*12*). PD-linked mutations, located both within the kinase and at distal domains, share this common feature of increasing LRRK2 kinase activity (*13*).

Kinases are highly “druggable” proteins, with more than 80 kinase inhibitors now clinically approved worldwide (*14*). Given LRRK2’s status as a key, actionable therapeutic target for PD, considerable efforts have been made in recent years to develop high-affinity, highly specific LRRK2 kinase inhibitors. The first generation of selective LRRK2 inhibitors, such as LRRK2-IN-1, CZC-25146, and GNE-7915, was published in 2011 (*15*–*17*). In 2015, the next generation of LRRK2 inhibitors, which included PF-360 and MLi-2, was disclosed (*18*, *19*). These next-generation inhibitors have been transformative for the field, having high affinity for LRRK2 and exceptional selectivity profiles. MLi-2 is regarded as the gold standard chemical tool compound for LRRK2 inhibition and has contributed considerably to understanding LRRK2’s mechanism and function at the molecular, cellular, and organismal scales. Despite the excellent pharmacological properties of these published inhibitory compounds and two clinical trials (clinicaltrials.gov) for LRRK2 kinase inhibitors now in progress, no LRRK2-targeting kinase inhibitor has been approved for clinical use.

All LRRK2 kinase inhibitors published to date are type I inhibitors, which bind to the kinase active site and stabilize an active-like/closed conformation (Fig. 1B). In contrast, while broad-spectrum type II inhibitors including GZD-824 and Rebastinib efficiently target LRRK2 (*20*–*22*), there are currently no published highly selective type II LRRK2 inhibitors that could be used to study the effects of targeting the LRRK2 inactive conformation. Thus, generating LRRK2-specific type II kinase inhibitors is a critical need for probing the cellular function of LRRK2 and for the further development of LRRK2 therapeutics. Type II inhibitors stabilize the inactive/open kinase conformation (Fig. 1C), reaching into the far back pocket of the kinase active site and disrupting a critical salt-bridge by releasing the regulatory αC helix (*23*, *24*). These disruptions prevent the Y from the DYG motif in the LRRK2 kinase active site (or F in the DFG motif commonly found in other kinases) from docking into the back pocket and placing the D in its catalytically competent position (the “DYG in” conformation), instead placing the DYG into the “DYG out” conformation (Fig. 1C) (*23*, *24*). Recent cryo–electron microscopy (cryo-EM) structures from our group and others of LRRK2 bound to selective type I and nonselective type II inhibitors have revealed the key structural differences in engaging the two main classes of kinase inhibitor, paving the way for the rational design of new inhibitory compounds (*21*, *25*).

Recent studies have highlighted the significance of these contrasting conformational changes within the LRRK2 kinase domain in terms of LRRK2 activation and subcellular localization (*22*, *26*–*28*). While the vast majority of LRRK2 is predicted to be cytosolic, LRRK2 activation requires it to be recruited to membranes where its substrates, Rab guanosine triphosphatases (GTPases), are located (*29*–*31*). In addition, under certain conditions, LRRK2 forms filamentous associations with microtubules (*32*, *33*) that block the movement of dynein and kinesin motor proteins in vitro (*22*). Type I inhibitors enhance LRRK2’s ability to block motor motility, whereas type II inhibitors can restore motor protein movement in the presence of LRRK2 (*22*). Type I inhibitors also induce dephosphorylation of the LRRK2 biomarker phosphorylation site S935, a response not elicited by type II inhibitors (*34*). Potentially pathogenic histopathological changes to the lungs of mice and nonhuman primates have also been described upon LRRK2 depletion or pharmacological inhibition with type I inhibitors (*35*, *36*). Whether these effects are related to the specific mode of action of type I kinase inhibitors is unknown, but, together these data reinforce the importance of developing LRRK2-specific type II kinase inhibitors both as potential therapeutics against PD and as chemical probes to explore the functional significance of LRRK2’s different conformational states in cells and in vivo.

Here, we set out to design type II inhibitory compounds with high affinity for LRRK2 and significantly improved selectivity profiles compared to the available broad-spectrum type II kinase inhibitors that can act on LRRK2. We used a combinatorial chemistry approach for the compound design, integrating the hinge-binding properties of well-characterized and selective type I LRRK2 inhibitors with the binding mode of promiscuous type II inhibitors known to associate with LRRK2 (Fig. 1D). Here, to our knowledge, we present the development and characterization of the first LRRK2 type II kinase inhibitors, named RN277 and RN341, as cellular tools targeting the LRRK2 inactive state.

## RESULTS

### Compound design, synthesis, and evaluation

#### MLi-2 hybridization series

The type I kinase inhibitor MLi-2 is the gold standard chemical tool compound for LRRK2 due to its outstanding kinome selectivity and high affinity (picomolar range) (*19*). Therefore, to develop a LRRK2-targeting type II inhibitor, we chose to first use a hybrid design strategy (Fig. 1D) that incorporates the specificity and excellent pharmacokinetic properties of MLi-2 with type II allosteric DYG-out binding moieties of established, broad-spectrum type II inhibitors. Specifically, our design was based on combining the hinge-binding indazole of MLi-2 with the DYG-out pocket binding moieties from GZD-824 and Rebastinib, two broad-spectrum type II inhibitors known to bind LRRK2. Additionally, we screened a library of published type II inhibitors to identify those that bind to LRRK2^KW^ (a kinase and WD40 domain construct of LRRK2) with high affinity. Using differential scanning fluorimetry (DSF), we identified Foretinib as an additional broad-spectrum type II LRRK2 ligand (table S1), suitable for use as a parental type II inhibitor in our hybrid approach. This initial synthesis strategy using MLi-2 as the parental type I compound led to the generation of eight candidate LRRK2-directed hybrid type II inhibitors.

Next, we evaluated the potency and selectivity of these new compounds. We performed on-target screening by measuring the thermostability increase of LRRK2^KW^ in the presence of the new compounds using DSF (table S2). We also determined the in vitro median inhibitory concentration (IC_50_) values for each of the new compounds by purifying LRRK2^RCKW^ and measuring its kinase activity in the presence of our new inhibitors using the PhosphoSense activity assay from AssayQuant (table S2). Last, we estimated the kinome selectivity of each compound by measuring the thermostability increase of ~100 representative kinases in the presence of the compounds via DSF (table S2). Only one compound (**52**; table S2) of our first series of MLi-2–inspired hybrid compounds showed high thermal stabilization of LRRK2^KW^ and inhibited LRRK2 kinase activity. Compound **52** increased the melting temperature of LRRK2^KW^ (Δ*T*_m_) by 10 K, suggesting strong binding, and inhibited LRRK2 kinase activity with a sub-micromolar IC_50_ value. However, the selectivity of compound **52** was unfavorable, demonstrated by the stabilization of 27% of the 100 kinases that we screened in our initial DSF selectivity panel (table S2).

#### PF-360 hybridization series

Because most of the MLi-2–like hybrid type II inhibitors demonstrated inadequate LRRK2 binding, we exchanged the hinge binding moiety to the 7*H*-pyrrolo[2,3-*d*]pyrimidine, inspired by PF-360 (Fig. 1D), another type I LRRK2 binding compound with a favorable selectivity profile (*18*). This moiety was fused with the DYG-binding moieties of type II inhibitors GZD-824 and Foretinib to give rise to two potential LRRK2 inhibitors, **1** and **2**.

We evaluated the potency and selectivity of these two compounds using the assays previously described (Table 1). In terms of on-target binding, we found that compound **2** bound more strongly (higher ∆*T*_m_) to LRRK2^KW^ than compound **1** (Table 1). **2** also demonstrated strong inhibition of LRRK2 kinase activity with an IC_50_ value of 185 nM (Table 1). However, as observed for the MLi-2–inspired compound **52**, the selectivity values of both **1** and **2** were still unfavorable (Table 1). Given the potent on-target binding and inhibitory activity of compound **2**, we next synthesized a larger set of PF-360–Foretinib–inspired inhibitors.

**Table 1.** First PF-360 hybridization series. Compound structures, thermal shift data, and in vitro IC_50_ values of PF-360–inspired type II hybrid compounds. The selectivity was calculated as the number of kinases showing a thermal shift greater than 5 K divided by the total number of kinases screened. NA, not applicable.

	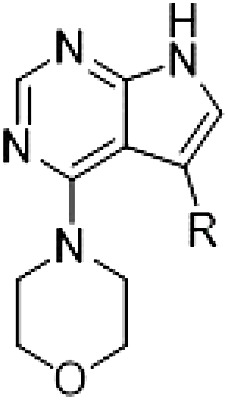			
Compound		∆*T*_m_ (K)	IC_50_ (nM)	*S* (5 K)
**1**	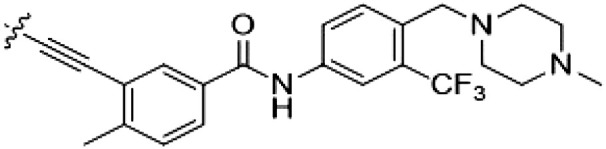	9.5	756 ± 20	0.20
**2**	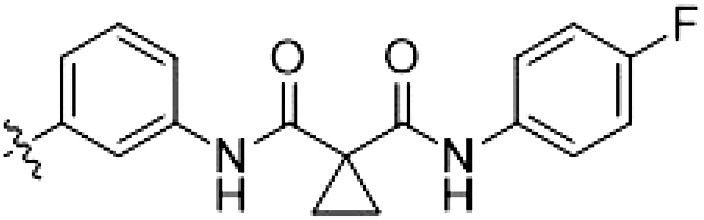	10.8	185 ± 10	0.28
**3**	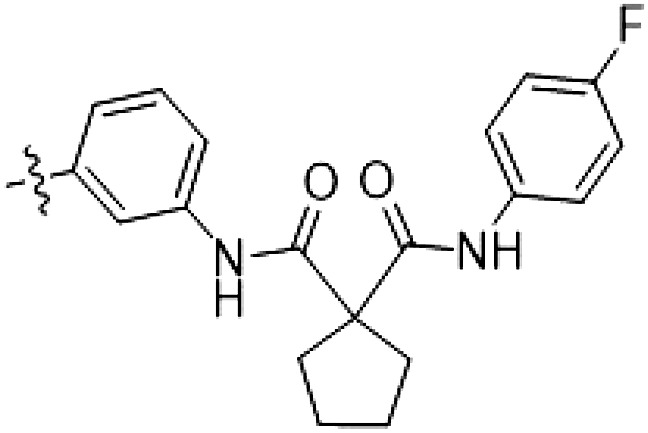	6.0	694 ± 1	NA
**4**	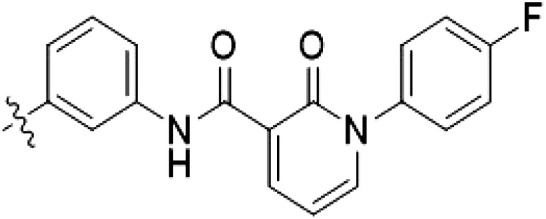	13.0	167 ± 4	0.33
**5**	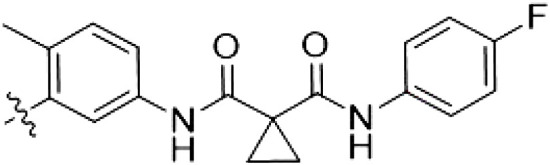	1.8	9580 ± 565	NA
**6**	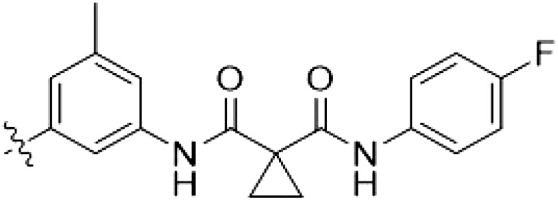	10.0	2145 ± 76	0.13
**7**	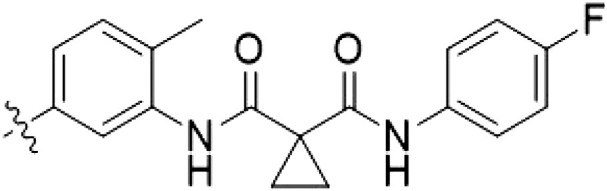	7.8	10,500 ± 1022	NA
**8**	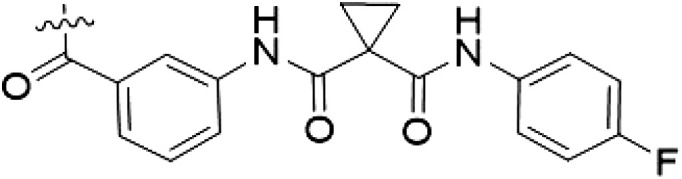	7.0	912 ± 141	NA
**9**	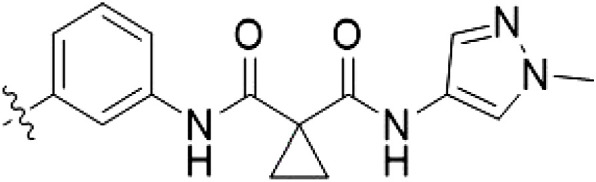	4.0	2110 ± 1189	NA
**10**	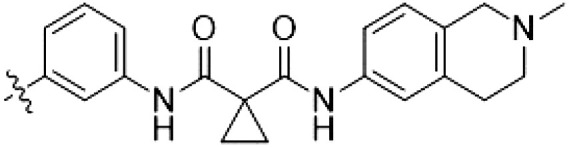	4.3	3180 ± 134	NA
**11**	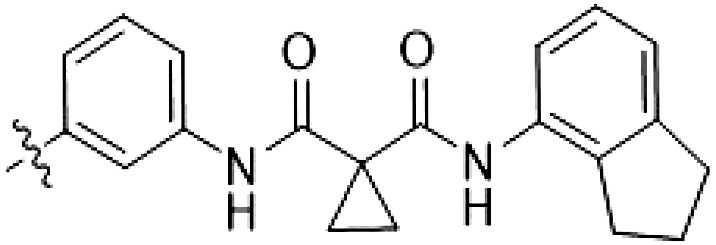	9.2	989 ± 32	0.15

In this set of **2**-derived analogs, major changes to the inhibitor geometry were introduced. These changes led to weaker inhibition or less selective compounds (Table 1), and we therefore focused the structure-activity relationship analysis on compound **2**. We hypothesized that the terminal phenyl ring of **2** would be positioned in the far back pocket of the kinase active site, similar to the crystal structures of the parental inhibitor Foretinib with other kinases [Protein Data Bank (PDB): 6I2Y and 5IA4]. This region of the binding pocket is less conserved in kinases compared to the adenosine 5′-triphosphate (ATP) binding site, which may allow for improved selectivity by introducing diverse moieties at this position (*23*, *24*). We systematically alternated the substitution pattern of the terminal phenyl ring by synthesizing 16 new hybrid type II inhibitors.

By evaluating the potency and selectivity of these compounds (Table 2) we found that the fluorine in para position (R^3^) of the phenyl ring is beneficial for LRRK2 binding (**12** to **16**). The addition of substituents in meta position (R^2^/R^4^) (**17** to **21**) was well accepted, with IC_50_ values in the low nanomolar range (Table 2). With a bromine in meta position (**19**), the stabilization of LRRK2^KW^ increased. The addition of a fluorine substituent in ortho position (R^1^) (**23**) was accepted and the selectivity increased markedly to *S* (5 K) = 16%. We therefore combined more potent meta substitutions (R^2^/R^4^) with more selective ortho (R^1^) substituted derivatives to produce two regioisomers, **25** and **26** (named RN222). This new compound, RN222 (**26**), showed both increased inhibition of LRRK2^RCKW^ kinase activity and improved selectivity [IC_50_ = 134 nM and *S* (5 K) = 14%; Table 2].

**Table 2.** Second PF-360 hybridization series. Compound structures, thermal shift data, and in vitro IC_50_ values of compound **2**–derived hybrid type II inhibitors. The selectivity was calculated as the number of kinases showing a thermal shift greater than 5 K divided by the total number of kinases screened.

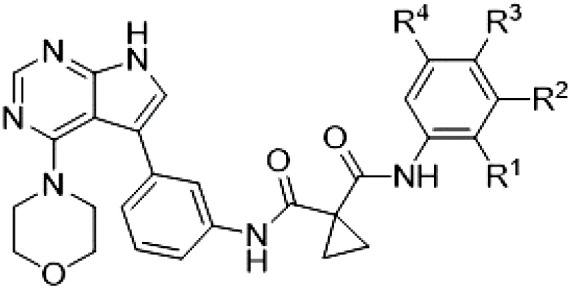							
Compound	R^1^	R^2^	R^3^	R^4^	∆*T*_m_ (K)	IC_50_ (nM)	*S* (5 K)
**2**	H	H	F	H	10.8	185 ± 10	0.28
**12**	H	H	H	H	9.3	350 ± 25	0.26
**13**	H	H	Cl	H	9.5	717 ± 14	0.37
**14**	H	H	Me	H	9.1	156 ± 33	0.22
**15**	H	H	Et	H	9.1	960 ± 33	0.23
**16**	H	H	OMe	H	4.3	1100 ± 217	0.10
**17**	H	F	F	H	10.3	253 ± 33	0.32
**18**	H	Cl	F	H	12.3	152 ± 44	0.36
**19**	H	Br	F	H	14.0	264 ± 19	0.31
**20**	H	Me	F	H	13.3	110 ± 11	0.42
**21**	H	OMe	F	H	9.3	331 ± 59	0.28
**22**	H	*t*Bu	H	H	12.0	886 ± 63	0.22
**23**	F	H	F	H	9.2	159 ± 0.2	0.16
**24**	Me	H	F	H	8.5	1190 ± 231	0.12
**25**	F	H	F	Br	9.0	818 ± 56	0.10
**26** (RN222)	F	Br	F	H	10.7	134 ± 5	0.14
**27**	H	Br	H	F	10.2	1960 ± 652	0.09

However, despite substantial improvements compared to our earlier series of hybrid type II inhibitors, the selectivity of **26** (RN222) still fell short of our criteria for a LRRK2-specific compound. Therefore, another back pocket type II binding motif was tested. Because the PF-360 hinge binding moiety with the linking phenyl ring in position 5 of the 7*H*-pyrrolo[2,3-*d*]pyrimidine provided successful on-target association with LRRK2^RCKW^ across most compounds in our second series of inhibitors, we kept this hinge binding scaffold and combined it with the DYG-binding motif of a different type II inhibitor, Rebastinib.

#### PF-360–Rebastinib hybrid series

The initial compound **28** obtained from this chemical series, named RN129, outperformed all previously designed and tested hybrid type II inhibitors both in stabilizing LRRK2^KW^ and in inhibiting LRRK2 kinase activity (IC_50_ = 59.7 nM) (Table 3). Unfortunately, RN129 (**28**) was not only, by far, the most potent LRRK2 inhibitor designed using our hybridization approach, but also the most promiscuous one, showing high thermal stabilization of 49% of the kinases screened.

**Table 3.** Second PF-360–Rebastinib hybrid series. Compound structures, thermal shift data, and in vitro IC_50_ values of PF-360–Rebastinib inspired type II hybrid compounds. The selectivity was calculated as the number of kinases showing a thermal shift greater than 5 K divided by the total number of kinases screened. NA, not applicable; ND, not determined.

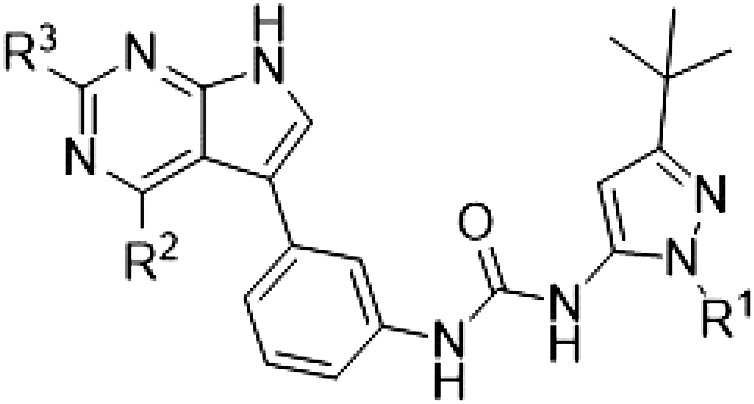						
Compound	R^1^	R^2^	R^3^	∆*T*_m_ (K)	IC_50_ (nM)	*S* (5 K)
**28** (RN129)	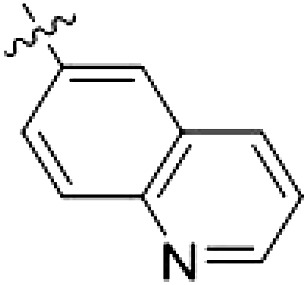	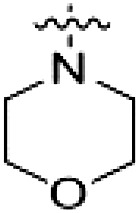	H	20.0	59.7 ± 2.9	0.49
**29**	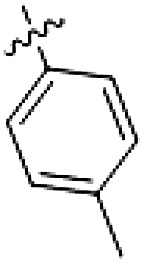	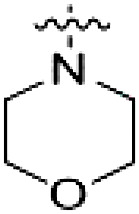	H	21.0	20.2 ± 0.01	0.36
**30** (RN277)	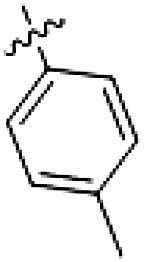	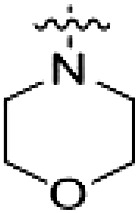	CI	18.0	135 ± 2	0.10
**31**	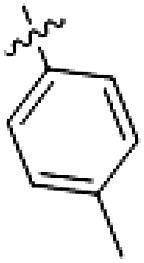	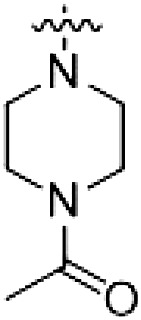	CI	18.0	82.4 ± 19	0.15
**32** (RN341)	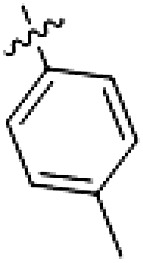	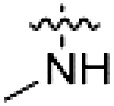	SEt	19.9	296 ± 39	0.10
**33**	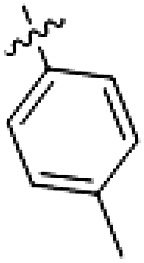	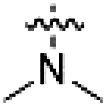	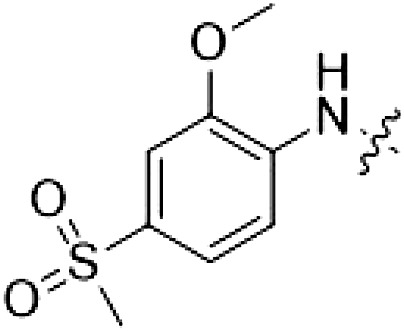	1.0	ND	NA
**34**	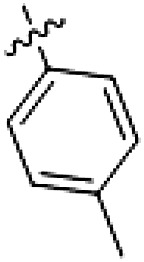	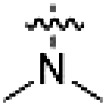	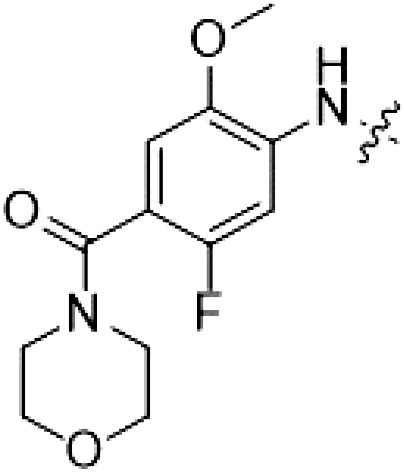	0.0	2820 ± 1703	NA
**35**	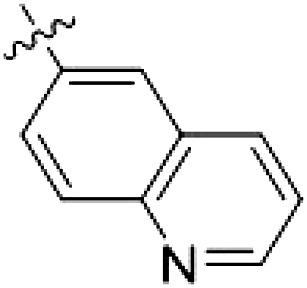	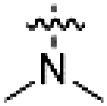	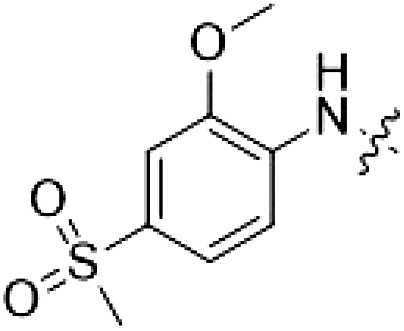	1.5	ND	NA
**Compound**	**–**	**R** ^**2**^	**R** ^**3**^	**∆*T***_**m**_ **(K)**	**IC**_**50**_ **(nM)**	***S* (5 K)**
**36**	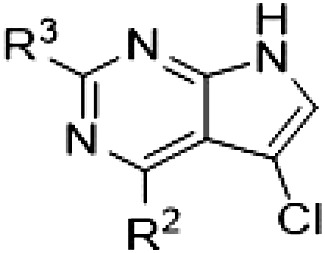	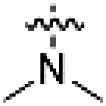	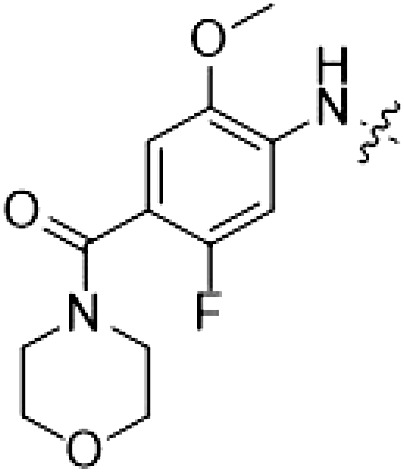	16.0	4.96 ± 0.11	0.14
**37**	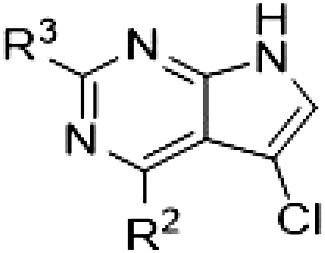	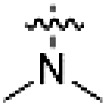	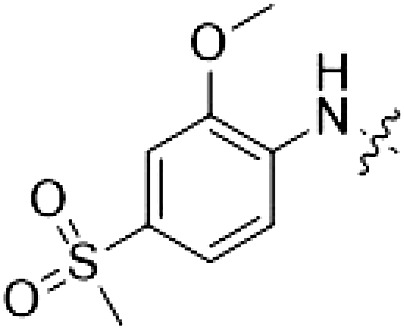	12.5	7.21 ± 2.1	0.05

**Fig. 2.**
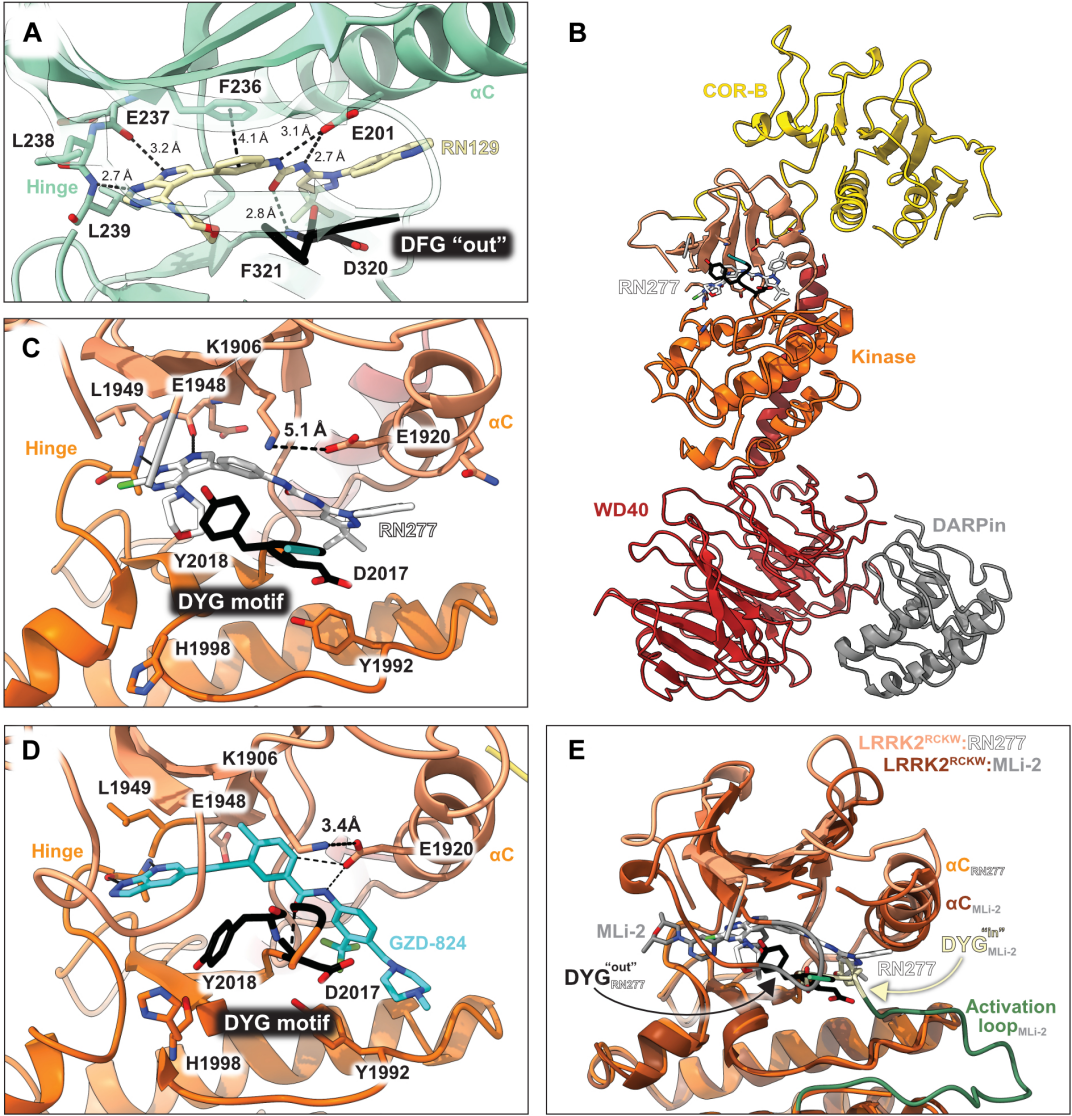
Co-crystal structure of RN129 bound to CLK3 and cryo-EM structure of RN277 bound to LRRK2^RCKW^. (**A**) The co-crystal structure of RN129 (**28**) with CLK3 highlighting the type II binding mode and interactions between the protein and inhibitor (PDB: 9EZ3). (**B**) Ribbon diagram of the atomic model of LRRK2^RCKW^:RN277:E11 DARPin complex (PDB: 9DMI) built into the cryo-EM map. (**C** and **D**) Close-ups of the active sites of the cryo-EM structures of LRRK2^RCKW^:RN277 (C) and LRRK2^RCKW^:GZD824 (PDB: 8TZE) (D). (**E**) Superposition of the atomic model of LRRK2^RCKW^:RN277:E11 DARPin complex (in lighter shades) and our previously published structure of a LRRK2^RCKW^:MLi-2:E11 DARPin complex (PDB: 8TXZ) (in darker shades). Only the kinase domains, which were aligned on their C-lobes, are shown. Major features of the kinase, including those that are indicators of type I and type II inhibitor binding, are shown.

To elucidate the binding mode of RN129 (**28**) and to inform future design strategies, we obtained a co-crystal structure of RN129 (**28**) with one of its potent off-targets, CDC-like kinase 3 (CLK3), a kinase that has been crystallized routinely in our group (Fig. 2). Our structure revealed that RN129 (**28**) has a canonical type II binding mode that has not been described for CLK3 previously. As expected, the 7*H*-pyrrolo[2,3-*d*]pyrimidine moiety of RN129 (**28**) formed two hydrogen bonds with the hinge of CLK3. The linking phenyl ring formed a π-π interaction with the phenylalanine F236. The urea was located between the αC helix and the DFG motif, with the bulky tail of the molecule extending into the far back pocket. The hinge of CLK3 contains a leucine residue (L238) that is also present in LRRK2, whereas most human kinases have larger phenylalanine or tyrosine residues at this position. Therefore, we hypothesized that developing the inhibitor toward this residue would improve selectivity. Because RN129 (**28**) has a large molecular weight of 588 Da, we aimed to reduce the molecular weight of the inhibitor before specifically targeting it toward the leucine within the hinge region. Instead of the quinoline in the far back pocket, we installed a toluene (**29**), which resulted in slight improvements in potency and selectivity compared to RN129 (**28**) (Table 3). Next, a chlorine atom was installed at position 2 (R^3^) of the 7*H*-pyrrolo[2,3-*d*]pyrimidine hinge binder to produce hybrid compound RN277 (**30**). Using the assays previously described we demonstrated that this compound was markedly more selective [*S* (5 K) = 10%] than its predecessors while maintaining excellent on-target affinity with ∆*T*_m_ = 18 K and IC_50_ = 135 nM (Table 3).

**Fig. 3.**
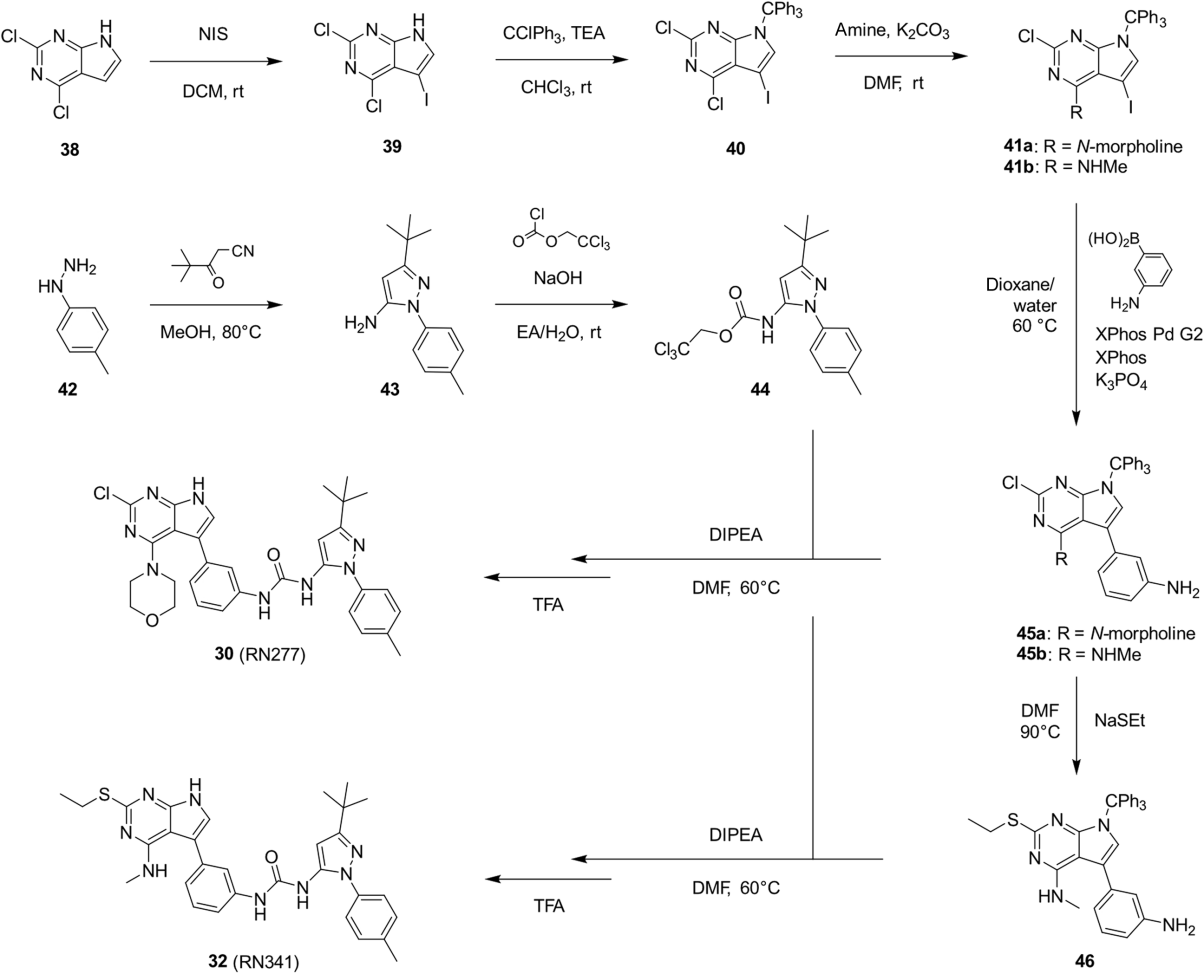
Synthesis of RN277 and RN341. The convergent synthesis route of compounds **30** (RN277) and **32** (RN341). The detailed procedures and analytics are shown in the supplementary information. rt, room temperature; NIS, *N*-iodosuccinimide; DCM, dichloromethane; TEA, triethylamine; DMF, dimethylformamide; XPhos, dicyclohexyl[2′,4′,6′-tris(propan-2-yl)[1,1′-biphenyl]-2-yl]phosphane; EA, ethyl acetate; DIPEA, *N*,*N*-diisopropylethylamine; TFA, trifluoracetic acid.

The high selectivity of RN277 (**30**) caused by the introduced chlorine in 2-position (R^3^) of the 7*H*-pyrrolo[2,3-*d*]pyrimidine was exploited by exchanging R^3^ with more space demanding moieties. Concurrently, the morpholine at the heterocycles 4-position was replaced by (di)methylamines to save on the inhibitors molecular weight. Our resulting lead compound, the hybrid type II inhibitor **32** (RN341, IC_50_ = 296 nM), was designed with a medium sized thiol ether residue at the 7*H*-pyrrolo[2,3-*d*]pyrimidine 2-position (R^3^). This compound maintained good on-target affinity (although slightly decreased compared to RN277) with high thermal stabilization of LRRK2^KW^ (∆*T*_m_ = 20 K) and an improved selectivity profile. RN341 (**32**) stabilized the same number of screened off-target kinases as RN277 [**30**, *S* (5 K) = 10%], but the degree of thermal stabilization of off-targets was lower (Table 3 and data S1). Together, DSF data, IC_50_ values, and observed LRRK2 selectivity of RN277 (**30**) and RN341 (**32**) indicate that both inhibitors have potential as tool compounds for specifically targeting and inhibiting LRRK2 kinase activity. The synthesis of both derivatives is outlined in Fig. 3.

### RN277 is a type II inhibitor of LRRK2

To address how the new type II inhibitor RN277 (**30**) binds to the kinase domain of LRRK2, we determined its structure by single particle cryo-EM, using purified recombinant LRRK2^RCKW^. The sample was prepared in the presence of guanosine diphosphate (GDP) as a ligand for the GTPase (ROC) domain, as we had done previously (*21*, *22*, *28*, *37*). We solved this structure bound to a LRRK2-specific designed ankyrin repeat protein (DARPin) (*37*). This small protein binds specifically to the WD40 domain of LRRK2 and decreases the preferential orientation of un-ligated LRRK2 on cryo-EM grids, improving particle distribution of LRRK2^RCKW^ (*37*). The map resolution allowed us to distinguish the molecular details of the kinase, especially in its active site. The map had an overall resolution of 3.3 Å, with local resolution reaching 3 Å around the kinase active site (fig. S1 and S2, and table S3). We then performed two focused refinements on different parts of the map: One comprised the ROC and COR-A domains, and the other comprised the COR-B, kinase, and WD40 domains. We built molecular models into each of these locally refined maps, which had better resolutions than the corresponding portion of the global map, and combined them into the final model (Fig. 2, B and C).

Our structure of LRRK2^RCKW^ bound to RN277 (**30**) revealed features of a type II inhibitor binding mode, including an open kinase conformation with the catalytic triad DYG motif “out” and a broken R-spine, and a disordered activation loop (Fig. 2, B and C). However, we also identify some features that differ from our previously reported structure of LRRK2^RCKW^ bound to the broad-spectrum type II inhibitor GZD-824 (*21*). First, the distance between K1906 and E1920 is 5.1 Å in the RN277 (**30**) structure, while it is 3.4 Å in the GZD-824 structure (Fig. 2, C and D). Second, there was no well-defined density for the G-rich loop in the N-lobe of the kinase in the RN277 (**30**) structure, while this motif was well resolved in the GZD-824 map. This suggests that the loop is flexible when LRRK2^RCKW^ is bound to RN277 (**30**). Third, the Y2018 of the DYG triad showed different conformations in the two structures (Fig. 2, C and D). Overall, our cryo-EM structure validated the type II inhibitor binding mode of our new LRRK2-targeting compound RN277 (**30**) and identified features that differed from the interaction observed in the structure of GZD-824 with LRRK2.

### Kinome selectivity profiling of RN341

**Fig. 4.**
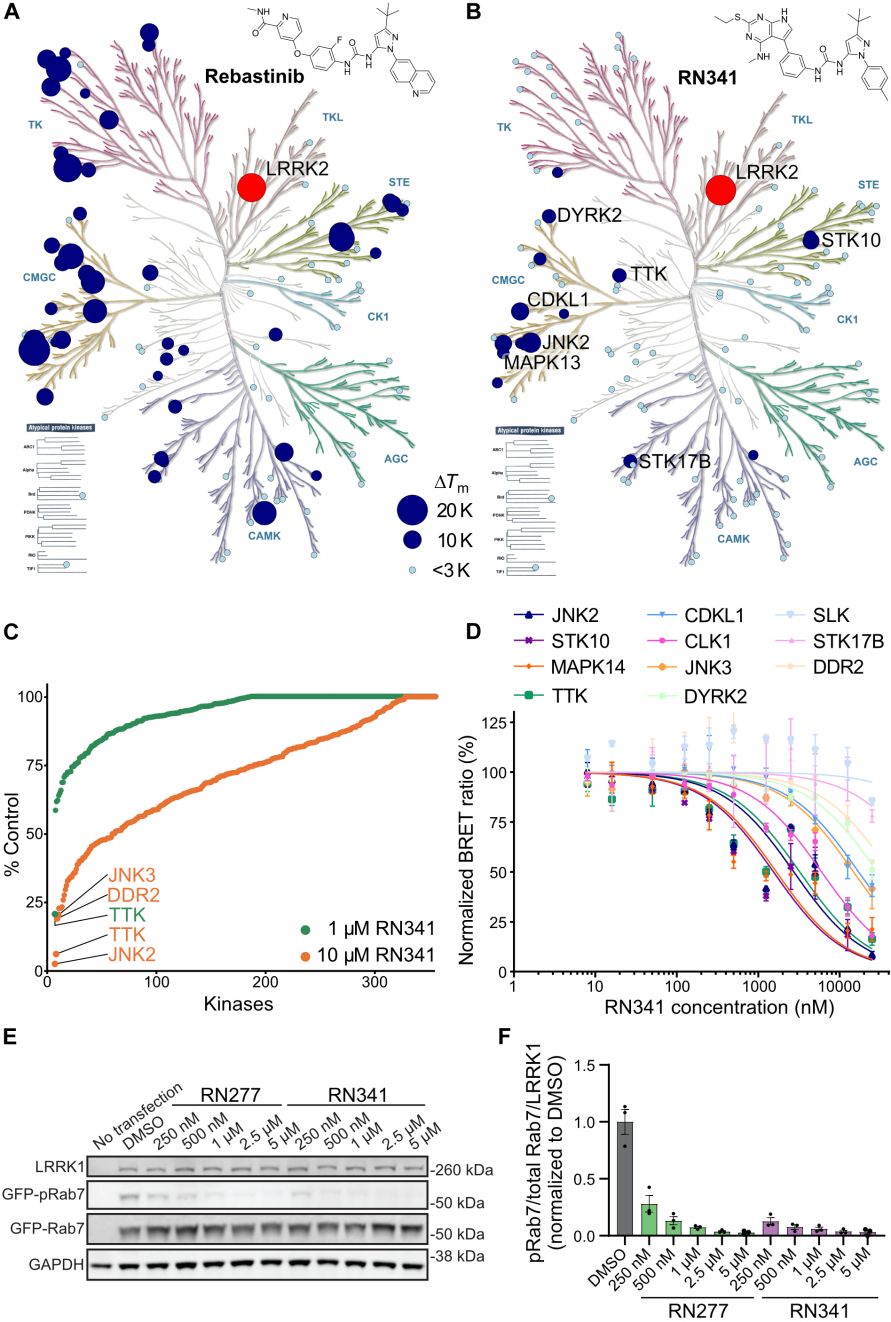
Kinome selectivity of RN341. (**A**) Kinome phylogenetic tree, with 96 kinases screened in the DSF assay against Rebastinib highlighted in blue or light blue. The 18.5 K ∆*T*_m_ shift of LRRK2^KW^ is highlighted in red. For all screened kinases, the bubble size and color correlates with the degree of ∆*T*_m_ shift, as indicated in the legend. (**B**) Kinome phylogenetic tree, with 103 kinases screened in the DSF assay against RN341 highlighted in blue. The 20-K ∆*T*_m_ shift of LRRK2^KW^ is highlighted in red. The bubble size or color for each kinase correlates with the ∆*T*_m_ shifts, as indicated in the legend (as in A). Kinases with ∆*T*_m_ > 6 K are labeled. (**C**) Waterfall plots of the ReactionBiology ^33^PanQinase screen of RN341 at 1 and 10 μM against 350 wild-type kinases. Kinases with decreased activity in the presence of RN341 to <22% of the control value are labeled. (**D**) Off-target validation from both screens via in cellulo nanoBRET assay in two biological replicates, error bars ± SD, EC_50_ (JNK2) = 2.7 μM, EC_50_ (STK10) = 1.5 μM, EC_50_ (MAPK14) = 1.7 μM, EC_50_ (TTK) = 3.2 μM, EC_50_ (CDKL1) = 17 μM, EC_50_ (CLK1) = 6.0 μM, EC_50_ (JNK3) = 15 μM, EC_50_ (DYRK2) ≥ 20 μM, EC_50_ (SLK) > 20 μM, EC_50_ (DDR2) > 20 μM, and EC_50_ (STK17B) ≥ 20 μM. (**E**) Representative immunoblot from 293T cells transiently co-transfected with LRRK1 and its substrate GFP-Rab7 before treatment with a dilution series of RN277 and RN341. Lysed cells were immunoblotted for LRRK1, GFP-Rab7, phospho-Rab7 (pS72), and GAPDH. (**F**) Quantification of the GFP-pRab7/GFP-Rab7/LRRK1 ratio from three independent Western blots. Statistical analysis performed using one-way analysis of variance (ANOVA) with Tukey’s multiple comparisons of means. *P* < 0.0001 for all inhibitor concentrations versus DMSO; error bars ± SEM.

As mentioned, the DSF selectivity screens of RN277 (**30**) and RN341 (**32**) showed the same number of kinases stabilized (>5 K) by both compounds. However, the degree of stabilization favors RN341 (**32**) as the more selective hybrid type II inhibitor (data S1). To understand how the selectivity of RN341 (**32**) for LRRK2 increased compared to commercially available type II inhibitors, we measured the DSF thermal stabilization of its broad-spectrum mother compound, the type II kinase inhibitor Rebastinib. The DSF selectivity screen for Rebastinib (Fig. 4A) revealed that 37 kinases were stabilized (>5 K) across almost all kinase families. In contrast, RN341 (**32**) showed only Δ*T*_m_ values of >5 K for 10 kinases, demonstrating an improved selectivity profile (Fig. 4B). The kinome selectivity of RN341 (**32**) was further characterized in a ^33^PanQinase activity assay screen of 350 kinases (ReactionBiology) at two different inhibitor concentrations (1 and 10 μM). The waterfall plot of this screen (Fig. 4C) showed that, even at the high concentration of 10 μM, for RN341 (**32**), only 10 kinases were inhibited with less than 30% residual activity remaining. At 1 μM RN341 (**32**), only threonine tyrosine kinase (TTK) showed inhibition less than 50% of control activity. Unexpectedly, RN341 (**32**) was not detected as a LRRK2 inhibitor in the ^33^PanQinase screen. LRRK2 is a large and unstable protein in vitro, and it is likely that the kinase was inactive in this selectivity panel. We confirmed the potent activity of RN341 (**32**) on LRRK2 in multiple orthogonal assay formats. The potential major off-target kinases (with ∆*T*_m_ > 5 K or % control kinase activity < 22% at 10 μM RN341) from both the DSF-screen and the ^33^PanQinase screen were validated in dose-response assays using a cellular nanoBRET assay (Fig. 4D) (*38*). The determined EC_50_ values of RN341 (**32**) indicate that of the 12 off-target kinases identified, only 4 (STK10, MAPK14, JNK2, and TTK) were inhibited by RN341 (**32**) with an EC_50_ in the low micromolar range (<5 μM).

The most closely related kinase to LRRK2 in the human genome is LRRK1 (*39*). LRRK1 shares a similar domain structure to LRRK2, lacking only the N-terminal ARM domain. Mutations in LRRK1 are linked to the rare bone diseases osteopetrosis and osteosclerotic metaphyseal dysplasia (*40*–*42*). Given the similarities between LRRK2 and LRRK1 and because LRRK1 was not included in the kinase panels initially used to determine inhibitor specificity, we tested the activity of our top compounds RN277 (**30**) and RN341 (**32**) toward LRRK1. To determine whether RN277 and RN341 could inhibit LRRK1 kinase activity in cells, we transfected 293T cells with LRRK1 and its substrate Rab7. We assessed the level of Rab7 phosphorylation by Western blotting. Our in-cell assays showed that both compounds display a strong inhibitory effect on LRRK1 kinase activity (Fig. 4, E and F). Together, our selectivity screens indicated that our lead compounds are potent and selective inhibitors of LRRKs, outperforming the selectivity of commercially available broad spectrum type II inhibitors used in our design strategy.

### In vitro and in cellulo inhibition of LRRK2 kinase activity by RN277 and RN341

**Fig. 5.**
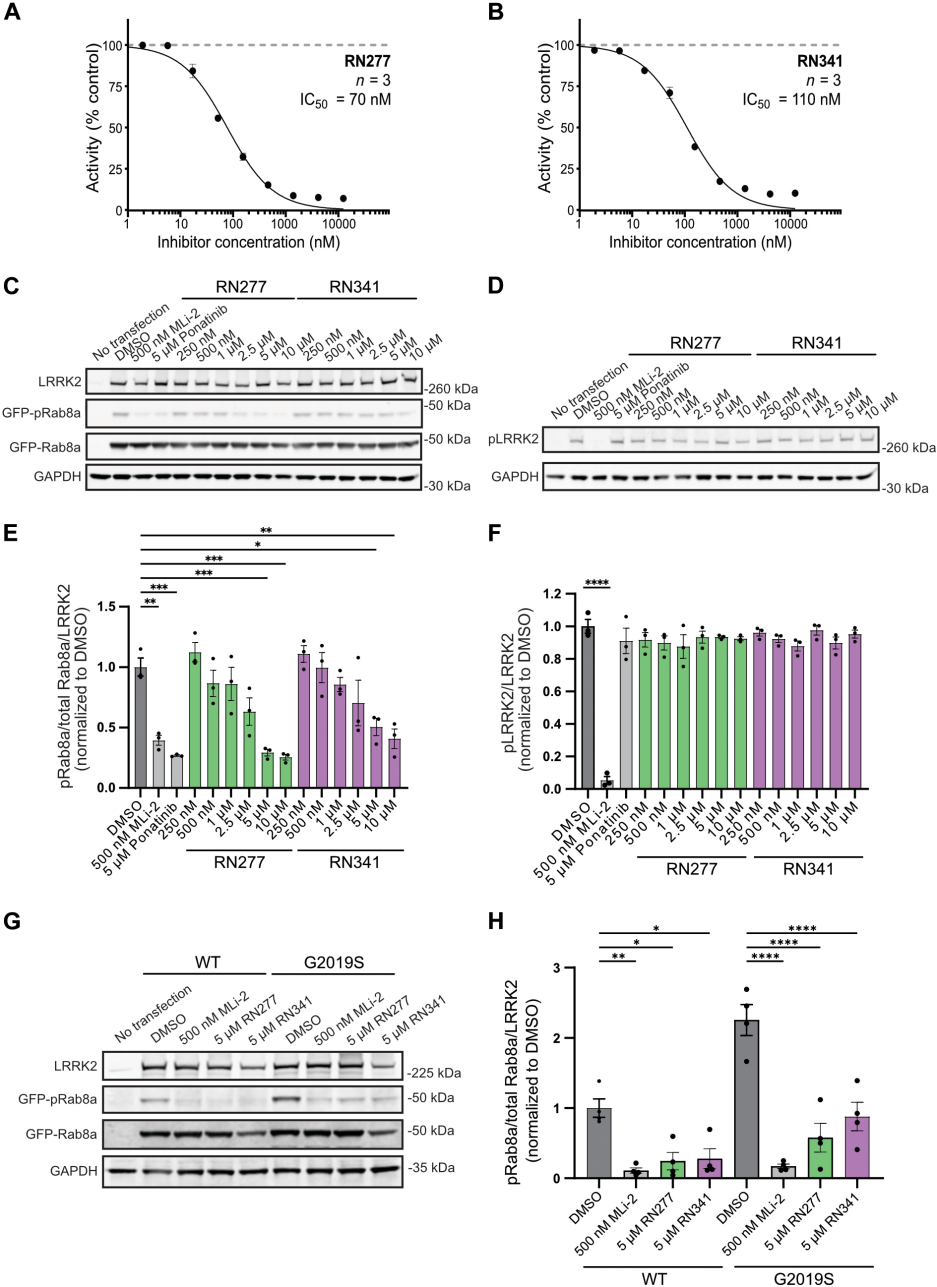
Inhibition of LRRK2’s phosphorylation of Rab8a in vitro and in cellulo. (**A** and **B**) Dose-response curve of RN277 (A) and RN341 (B) inhibiting LRRK2^RCKW^-mediated phosphorylation of Rab8a. Activity was calculated as the percentage (%) of phosphorylated Rab8a versus non-phosphorylated Rab8a detected in the presence of different concentrations of RN277/RN341. (**C**) Representative immunoblot from 293T cells transiently co-transfected with LRRK2 and GFP-Rab8a, treated with the indicated inhibitors. Lysed cells were immunoblotted for LRRK2, GFP-Rab8a, phospho-Rab8a (pT72), and GAPDH. (**D**) Sample from (C) run separately under identical conditions and immunoblotted for phospho-S935 LRRK2 and GAPDH. (**E**) Quantification of the GFP-pRab8a/GFP-Rab8a/LRRK2 ratio from three independent immunoblots (C). Statistical analysis performed using one-way ANOVA with Tukey’s multiple comparisons of means. **P = 0.0049, DMSO versus MLi-2; ***P = 0.0004, DMSO versus Ponatinib; ***P = 0.0006, DMSO versus 5 μM RN277; ***P = 0.0003, DMSO versus 10 μM RN277; *P = 0.0406, DMSO versus 5 μM RN341; **P = 0.0065, DMSO versus 10 μM RN341; error bars ± SEM. (**F**) Quantification of the pS935 LRRK2/LRRK2 ratio (run under identical conditions on separate blots) from three independent immunoblots (D). Statistical analysis performed using one-way ANOVA with Tukey’s multiple comparisons of means. ****P < 0.0001 for all conditions versus MLi-2; error bars ± SEM. (**G**) Representative immunoblot from 293T cells transiently co-transfected with GFP-Rab8a and either GFP-11 tagged wild-type (WT) or GFP-11 tagged G2019S LRRK2, treated with the indicated inhibitors. Lysed cells were immunoblotted for LRRK2, GFP-Rab8a, phospho-Rab8a (pT72), and GAPDH. (**H**) Quantification of the GFP-pRab8a/GFP-Rab8a/LRRK2 ratio from four independent immunoblots (G). Statistical analysis performed using one-way ANOVA with Tukey’s multiple comparisons of means. **P = 0.0077, WT LRRK2 DMSO versus MLi-2; *P = 0.0324, WT LRRK2 DMSO versus 5 μM RN277; *P = 0.0461, WT LRRK2 DMSO versus 5 μM RN341; ****P < 0.0001 for all inhibitor treatments versus G2019S LRRK2 DMSO; error bars ± SEM.

The best characterized physiological substrates of LRRK2 are a subset of small Rab GTPases (*43*). To investigate the ability of RN277 (**30**) and RN341 (**32**) to inhibit LRRK2 kinase activity toward its physiological substrates in vitro, we incubated a dilution series of the compounds with recombinant LRRK2^RCKW^ and Rab8a and quantified Rab8a phosphorylation (pT72) by mass spectrometry. As anticipated, increasing quantities of each compound resulted in decreased Rab8a phosphorylation, indicative of inhibited LRRK2 kinase activity. RN277 (**30**) displayed a slightly better inhibitory effect (IC_50_ = 70 nM) compared to RN341 (**32**, IC_50_ = 110 nM) (Fig. 5, A and B).

Next, we determined whether RN277 (**30**) and RN341 (**32**) inhibited LRRK2 kinase–mediated phosphorylation of Rab8a in cells (Fig. 5, C and E). To do this, we co-transfected 293T cells with LRRK2 and green fluorescent protein (GFP)–Rab8a. Transfected cells were treated with increasing concentrations of RN277 and RN341, and the level of Rab8a phosphorylation was assessed by Western blotting. Toxicity did not appear to be an issue with either of these compounds, at any of the concentrations used.

Both compounds reduced phosphorylation of Rab8a in a dose-dependent manner (Fig. 5, C and E). In addition, we tested the effect of increasing concentrations of RN277 and RN341 on phosphorylation of the LRRK2 biomarker site S935. The ability to inhibit phosphorylation of LRRK2 substrate Rabs (such as Rab8a) without affecting the phosphorylation of LRRK2 biomarker site S935 is a known feature of nonspecific type II LRRK2 kinase inhibitors (*34*). In contrast to treatment with 500 nM MLi-2, which led to almost complete dephosphorylation of LRRK2 S935, none of the tested type II inhibitors (broad-spectrum kinase inhibitor Ponatinib or our top compounds RN277 and RN341) reduced the phosphorylation level at this site (Fig. 5, D and F).

Given the potential for type II LRRK2 kinase inhibitors to be used as therapeutics for PD, we also tested whether our new compounds could inhibit kinase activity of the common G2019S PD allele of LRRK2. In 293T cells transfected with GFP-Rab8a and either wild-type or G2019S LRRK2, RN277 (**30**) and RN341 (**32**) inhibited phosphorylation of Rab8a by both the wild-type and mutant kinase (Fig. 5, G and H).

### Type II inhibitors rescue LRRK2 inhibited kinesin motility in vitro

**Fig. 6.**
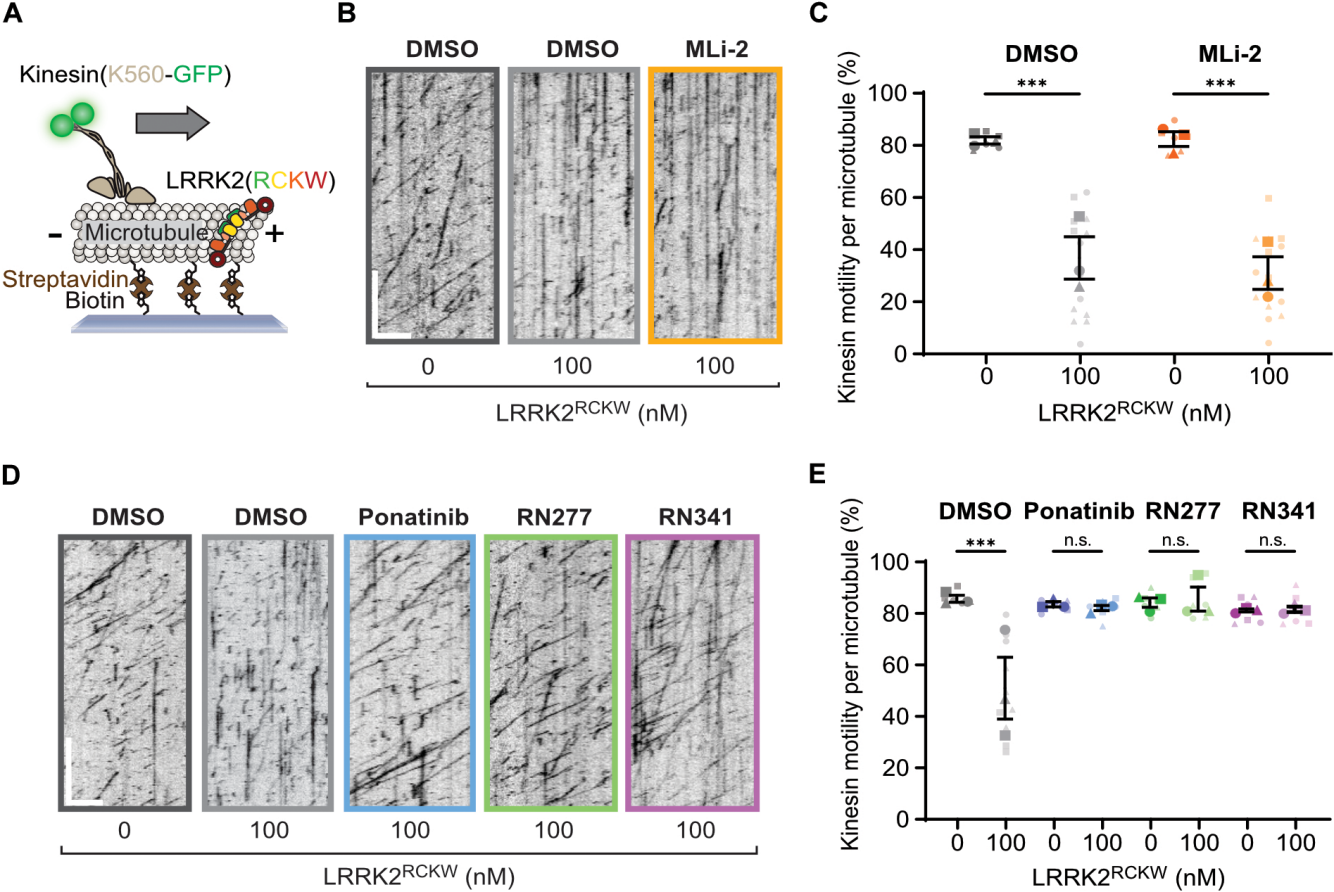
LRRK2-specific type II inhibitors RN277 and RN341 rescue kinesin motility in the presence of LRRK2^RCKW^. (**A**) Schematic of the single-molecule in vitro motility assay. (**B**) Example kymographs from single-molecule motility assays showing kinesin motility with DMSO or the type I inhibitor MLi-2 (5 μM) in the presence or absence of LRRK2^RCKW^. Scale bars, 5 μm (*x*) and 30 s (*y*). (**C**) Quantification of the percentage (means ± SEM) of motile events per microtubule as a function of LRRK2^RCKW^ concentration in the absence (DMSO) or presence of MLi-2 (5 μM). Three technical replicates were collected per condition, with data points represented as circles, triangles, and squares corresponding to single data points (microtubules) within each replicate. Statistical analysis was performed using the mean of each technical replicate; ***P = 0.0007, DMSO condition; ***P = 0.0003, MLi-2 condition, one-way ANOVA with Šidák’s multiple comparisons test within drug only. (**D**) Example kymographs from single-molecule motility assays showing kinesin motility with DMSO or the type II inhibitors Ponatinib, RN277, and RN341 (10 μM) in the presence or absence of LRRK2^RCKW^. Scale bars, 5 μm (*x*) and 30 s (*y*). (**E**) Quantification of the percentage (means ± SEM) of motile events per microtubule as a function of LRRK2^RCKW^ concentration in the absence (DMSO) or presence of type II inhibitors Ponatinib, RN277, and RN341 (10 μM). Three technical replicates were collected per condition, with data points represented as circles, triangles, and squares corresponding to single data points (microtubules) within each replicate. Statistical analysis was performed using the mean of each technical replicate; ***P = 0.0003, one-way ANOVA with Šidák’s multiple comparisons test within drug only.

Previous work showed that LRRK2^RCKW^ can associate with microtubules (*22*, *26*–*28*) and block dynein and kinesin motor protein movement in vitro (*22*). As demonstrated with nonspecific inhibitors in these studies, type II inhibitors, which stabilize the open kinase conformation, rescue motor protein motility, while type I inhibitors that stabilize the closed kinase form do not (*22*). Given our structural evidence that RN277 (**30**) stabilized the open kinase conformation of LRRK2 (Fig. 2, B and C), we hypothesized that our new type II compounds would prevent LRRK2 filamentation on microtubules and, therefore, relieve LRRK2^RCKW^-dependent inhibition of kinesin motility in single-molecule in vitro motility assays (Fig. 6A). As previously described (*22*, *28*), LRRK2^RCKW^ impedes kinesin motility (Fig. 6). The LRRK2-specific type I inhibitor MLi-2, which stabilizes the closed kinase conformation (*21*), did not rescue this effect (Fig. 6, B and C). In contrast, RN277 (**30**), RN341 (**32**), and the broad-spectrum type II kinase inhibitor Ponatinib rescued kinesin motility from the inhibition observed in the presence of LRRK2^RCKW^ (Fig. 6, D and E). Thus, our data showed that RN277 (**30**) and RN341 (**32**) displayed a key hallmark of type II kinase inhibitors, namely, an ability to rescue kinesin motility in the presence of LRRK2^RCKW^, likely due to stabilization of the open kinase conformation that prevents LRRK2 filamentation on microtubules.

## DISCUSSION

Here, we presented the development and characterization of new type II LRRK2 kinase inhibitors RN277 and RN341. The success of our structure-directed approach combining parental LRRK2-specific type I and broad-spectrum type II inhibitors demonstrated a general rational design strategy that can be further exploited to develop new type II inhibitors based on type I inhibitors with favorable pharmacological properties. Due to its interaction with the allosteric and often diverse DFG-out pocket, the type II binding mode had been initially described as a strategy for the development of more selective inhibitors. However, kinome-wide selectivity studies showed that type II DFG-out pockets are accessible in most kinases and that type II inhibitors are often more promiscuous than type I compounds (*23*, *44*). Despite the excellent selectivity of available type I inhibitors, the development of selective type II inhibitors inspired by the hinge binding moieties of type I inhibitors was a formidable medicinal chemistry effort. The PD field will benefit from our lead compounds, which will enable avenues for research and drug development that may avoid the on-target side effects reported for type I LRRK2 inhibitors (*45*).

Both RN277 and RN341 showed excellent potency and selectivity. Both compounds bound to LRRK2, increasing the thermostability of LRRK2^KW^ in DSF assays (Table 3), and inhibited the phosphorylation of Rab8a by LRRK2^RCKW^ in vitro with 70 and 110 nM IC_50_ values, respectively (Fig. 5). Both compounds also inhibited LRRK2 phosphorylation of Rab8a in cells (Fig. 5). In two kinome selectivity screens of 103 and 350 kinases, only four major off-targets were identified for RN341 (STK10, MAPK14, JNK2, and TTK) with cellular activity in the micromolar region (Fig. 4). This represents a markedly improved selectivity profile compared to the broad-spectrum LRRK2 type II inhibitors now available. Both RN277 and RN341 also potently inhibited LRRK1 in cells. Thus, our new compounds are highly selective for LRRK proteins, and our work paves the way for future medicinal chemistry studies focused on producing type II inhibitors that are specific for LRRK2 over LRRK1.

Both RN277 and RN341 displayed the key phenotypic and structural hallmarks of LRRK2 type II kinase inhibitors. Both compounds preserve phosphorylation at the biomarker S935 site on LRRK2 (Fig. 5), a phenomenon that was previously described for other broad spectrum LRRK2 type II inhibitors. This phenotype demonstrates a key difference in the downstream effects of type I and type II LRRK2 inhibition, with type I LRRK2 inhibitors inducing dephosphorylation at these sites (*34*). Although the underlying mechanism for these differences is not known, it is hypothesized that the conformational changes in LRRK2 induced by type I and type II inhibitors modulate the accessibility of LRRK2 biomarker sites to upstream phosphatases and/or kinases. Our new LRRK1/2-specific type II inhibitors represent key tools that could be used to assess the conformation-specific accessibility and association of LRRK2 with these upstream modifiers. Additionally, both RN277 and RN341 rescue the motility of kinesin on microtubules in the presence of LRRK2^RCKW^, an effect that is specific to type II inhibitors due to their promoting the open kinase conformation (*22*, *28*) (Fig. 6). An open question in the field is whether the increased size and number of cytosolic lamellar bodies in the lungs of nonhuman primates following repeated dosing with type I LRRK2 inhibitors (*35*) could result from LRRK2-induced blockage of microtubule-based intracellular transport. The future use and characterization of RN277 and RN341 should help in answering this critical question.

We also present a 3-Å resolution cryo-EM structure of LRRK2^RCKW^ bound to RN277 (Fig. 2). Given the structural and chemical similarity of RN277 and RN341, we anticipate that they will bind LRRK2 in a largely comparable manner. In the LRRK2^RCKW^:RN277 structure, we show that RN277 displays the typical binding mode of a type II inhibitor, including the positioning of the LRRK2 catalytic triad in the “DYG-out” position. However, we also observed conformational differences compared to the cryo-EM structure of LRRK2^RCKW^ bound to the broad-spectrum type II inhibitor GZD-824 (*21*). This makes the new chemotype of RN277 and RN341 interesting for future investigations into the conformation-dependent regulation of LRRK2 and for the design of additional LRRK2-targeting inhibitory compounds.

The LRRK1/2-selective type II inhibitors RN277 and RN341 represent a new set of tool compounds, which will open avenues for therapeutic development for PD and that harbor substantial research potential for uncovering the broader impact of using conformation-specific LRRK2 inhibitors in vivo and in cells.

## MATERIALS AND METHODS

### Compound synthesis and analysis

The synthesis schemes (figs. S3 to S8) and procedures including all intermediate analytics as well as the nuclear magnetic resonance (NMR) spectra, high-performance liquid chromatography (HPLC)/mass spectrometry (MS) analysis, and high-resolution mass spectrometry (HRMS) data of all hybrid type II inhibitors are shown in the Supplementary Materials. All biologically tested compounds exceeded 95% purity in HPLC ultraviolet spectra and showed clean NMR spectra, and the mass error of the high-resolution mass spectrum was below 5 parts per million. (The synthesis and structural characterization of the LRRK2-targeting compounds RN277 and RN341 is available at https://dx.doi.org/10.17504/protocols.io.36wgqnxzxgk5/v1.)

### Protein expression and purification from insect cells

The LRRK2^KW^ (RRID: Addgene_228879) or LRRK2^RCKW^ (RRID: Addgene_226783) expression constructs (table S4) encoding a Tobacco Etch Virus (TEV) cleavable N-terminal His_6_-Z-tag were expressed in SF9 insect cells (RRID:CVCL_0549) after baculoviral transfection as described previously (*21*). In brief, the virus was generated according to Bac-to-Bac expression system protocols (Invitrogen). For protein expression, cells were infected at a density of 2 × 10^6^ cells/ml with recombinant virus. Infected cells were cultured in serum-free Insect-XPRESS Medium (Lonza) at 27°C shaking with 90 rpm for 66 hours. After harvesting by centrifugation, the cell pellet was washed with phosphate-buffered saline (PBS) before sonication in lysis buffer [containing 20 mM Hepes (pH 7.4), 500 mM NaCl, 20 mM imidazole, 0.5 mM Tris(2-carboxyethyl)phosphine (TCEP), 5% glycerol, 20 μM GDP, and 5 mM MgCl_2_]. The lysate was clarified by ultracentrifugation for 1 hour at 100,000*g* at 4°C and loaded onto pre-equilibrated Ni–nitrilotriacetic acid (NTA) Sepharose (QIAGEN). After stringent washing with lysis buffer, the protein was eluted in lysis buffer supplemented with 300 mM imidazole. After reducing the NaCl concentration to 250 mM, the eluate was loaded onto a SP sepharose column (Cytiva) and eluted by applying a salt gradient ranging from 250 mM to 2.5 M. The fractions containing the protein of interest were pooled and N-terminal tags were removed with TEV protease incubation overnight. Cleaved tag, contaminating proteins, and uncleaved protein were removed by running a combination of SP sepharose and Ni-NTA column. Last, the protein was subjected to size exclusion chromatography (SEC) using a S200 16/200 column (GE Healthcare) in 20 mM Hepes (pH 7.4), 800 mM NaCl, 0.5 mM TCEP, 0.5% glycerol, 20 μM GDP, and 2.5 mM MgCl_2_ coupled to an AKTA Xpress system. (For a step-by-step protocol, see dx.doi.org/10.17504/protocols.io.14egn314pl5d/v1.)

### Protein expression and purification from bacteria

The Rab8A substrate, encoding a TEV cleavable N-terminal His_6_-tag (RRID: Addgene_228880; table S4) was expressed in *Escherichia coli* Rosetta cells (Novagen, 70954; www.sigmaaldrich.com/US/en/product/mm/70954?msockid=2313305006bb6050160c228007f861f2; EMD_BIO-70954). Cells were grown shaking at 37°C until optical density at 600 nm (OD_600_) of 0.8 was reached. After reducing temperature to 18°C protein expression was induced by adding isopropyl-β-d-thiogalactopyranoside (IPTG), and cells were incubated overnight shaking at 180 rpm. Cells were harvested by centrifugation. Ni-NTA purification was essentially carried out as described above. The eluate from Ni-NTA was dialyzed straight away overnight, while the N-terminal tags were removed by adding TEV protease. Contaminating protein, the cleaved tag and non-cleaved protein were removed by Ni-NTA before the protein was concentrated and subjected to SEC as described above. (For step-by-step protocol, see dx.doi.org/10.17504/protocols.io.261ge514yg47/v1.)

The GFP-tagged human kinesin construct (K560-GFP; RRID:Addgene_15219) (table S4) was purified from BL21-CodonPlus (DE3)-RIPL (Agilent, 230280; www.agilent.com/store/en_US/Prod-230280/230280; RRID:CVCL_M639) *E. coli*, as previously described (*46*). In brief, transformed *E. coli* cultures were grown at 37°C to OD_600_ of 0.6 to 0.8 before induction with 0.75 mM IPTG for 16 hours at 18°C. After harvesting by centrifugation, *E. coli* pellets were resuspended in lysis buffer [50 mM tris (pH 7.5), 250 mM sodium chloride, 1 mM magnesium chloride, and 20 mM imidazole (pH 7.4)] supplemented with 0.5 mM Mg-ATP, 10 mM β-mercaptoethanol, 1 mM Pefabloc, complete EDTA-free protease inhibitor cocktail tablet (Roche), and 1 ml of lysozyme (50 mg/ml) and lysed by sonication on ice. Lysates were clarified by centrifugation at 92,600*g* for 30 min at 4°C and incubated with Ni-NTA agarose beads (QIAGEN) for 1 hour at 4°C. Beads and lysate were then transferred to a gravity flow column and washed repeatedly in lysis buffer supplemented with 0.5 mM Mg-ATP, 10 mM β-mercaptoethanol, and 1 mM Pefabloc. After washing, protein was eluted in elution buffer [50 mM tris (pH 7.5), 250 mM sodium chloride, 1 mM magnesium chloride, and 250 mM imidazole (pH 7.4)] supplemented with 0.1 mM Mg-ATP, and 10 mM β-mercaptoethanol. The eluate was transferred to a PD-10 column (GE Healthcare) for desalting and buffer exchanged into BRB80 [80 mM K-Pipes (pH 6.8), 2 mM magnesium chloride, 1 mM EGTA] supplemented with 0.1 mM Mg-ATP, 0.1 mM dithiothreitol (DTT), and 10% sucrose. (For step-by-step protocol, see dx.doi.org/10.17504/protocols.io.4r3l2qr1xl1y/v1.)

### Compound screening by DSF

To characterize binding between small molecules and LRRK2^KW^, induced thermal stabilization was measured by DSF according to previously established protocols (*47*). In brief, LRRK2^KW^ was diluted to 4 μM in 20 mM Hepes (pH 7.4), 150 mM NaCl, 0.5 mM TCEP, 0.5% glycerol, 20 μM GDP, and 2.5 mM MgCl_2_, and SYPRO Orange was added (1:1000, MilliporeSigma). Inhibitor (10 μM) to be tested was added to a final sample volume of 20 μl per well of a 96-well plate (white, Starlab). Fluorescence was monitored with excitation and emission filters set at 465 and 590 nm during gradual heating (1 K/min) from 25° to 95°C in a MX3005P real-time polymerase chain reaction (PCR) instrument (Stratagene). Data were evaluated using MXPro3005 software (Stratagene, www.agilent.com/cs/library/usermanuals/public/70225.pdf, RRID:SCR_016375). The fluorescence was plotted against the temperature, and the melting point (*T*_m_) was determined as the minimum of the first derivative relative to the control. (For step-by-step protocol, see dx.doi.org/10.17504/protocols.io.kxygx3y6kg8j/v1.)

### Biochemical LRRK2 activity assay

The biochemical activity assay was based on the phosphorylation reaction between LRRK2^RCKW^ and the SOX-based substrate peptide (AQT0615, Assay Quant Technologies). A dilution series between 15 μM and 0.4 nM of 11 concentrations of the compounds was pipetted into white 384-well plates (Greiner 781207) as duplicates with an ECHO acoustic dispenser (Labcyte). Two wells per compound were used as 0% (without protein and compound) and 100% (without compound) control. The purified LRRK2^RCKW^ was diluted to 22 nM with the reaction buffer [50 mM Hepes buffer (pH 7.5), 10 mM MgCl_2_, 1% glycerol, 1 mM DTT, 0.2 mg/ml bovine serum albumin (BSA), 0.01% Tween 20, and 5 μM AQT0615], and 10 μl was added in each well with a multichannel pipette (Eppendorf). The 0% control wells were filled with 10 μl of pure reaction buffer. With the ECHO, 5 nl of 100 mM ATP was pipetted into each well, the plates were centrifuged at 1500*g* for 2 min, and the reaction was incubated at room temperature for 4 hours. The fluorescence of the SOX-peptide after 360-nm excitation was measured at 487-nm emission with a PHERAstar plate reader (BMG Labtech). The IC_50_ values were calculated from a nonlinear regression of the log[inhibitor] versus the normalized response with GraphPad Prism 8 (www.graphpad.com/, RRID:SCR_002798). (For step-by-step protocol, see dx.doi.org/10.17504/protocols.io.81wgbz8qngpk/v2.)

### DSF 100 kinase selectivity screen

Recombinant protein kinase domains with a concentration of 2 μM were mixed with a 20 μM compound solution in dimethyl sulfoxide (DMSO), 20 mM Hepes (pH 7.5), and 500 mM NaCl. SYPRO Orange (5000×, Invitrogen) was added as a fluorescence probe (1μl/ml). Subsequently, temperature-dependent protein unfolding profiles were measured using the QuantStudio 5 realtime PCR machine (Thermo Fisher Scientific, Waltham, MA, USA). Excitation and emission filters were set to 465 and 590 nm. The temperature was raised with a step rate of 3°C/min. Data points were analyzed with the internal software (Protein Thermal Shift Software v1.4, www.thermofisher.com/order/catalog/product/4466038) using the Boltzmann equation to determine the inflection point of the transition curve (*48*). Differences in melting temperature are given as Δ*T*_m_ values in degrees Celsius. Measurements were performed in duplicates. (For step-by-step protocol, see www.eubopen.org/sites/www.eubopen.org/files/attachments/2021/DSF-inhibitorScreening_v1.pdf).

### Co-crystallization with CLK3

CLK3 kinase domain (residues 127 to 484) with a TEV-cleavable N-terminal His6-tag (RRID: Addgene_38831; table S4) was transformed into BL21(DE3) cells. Bacteria were grown in Terrific Broth medium (Thermo Fisher Scientific) containing kanamycin (50 mg/ml). Protein expression was induced at an OD_600_ of 2 using 0.5 mM IPTG at 18°C for 12 hours. Cells were lysed by sonication in lysis buffer containing 50 mM Hepes (pH 7.5), 500 mM NaCl, 25 mM imidazole, 5% glycerol, 50 mM l-glutamic acid, 50 mM l-arginine, and 0.5 mM TCEP. After centrifugation, the supernatant was loaded onto a Nickel-Sepharose column (Thermo Fisher Scientific) equilibrated with 30 ml of lysis buffer. The column was washed with 60 ml of lysis buffer. Protein was eluted by an imidazole step gradient (50, 100, 200, and 300 mM). CLK3 was dialyzed against SEC buffer [50 mM Hepes (pH 7.5), 500 mM NaCl, 5% glycerol, 50 mM l-glutamic acid, 50 mM l-arginine, and 0.5 mM TCEP] overnight, and TEV protease was added to remove tag. After a reverse Ni-rebinding column to remove TEV protease and uncleaved protein, the protein was further purified by SEC using a S75 16/60 HiLoad column (GE Healthcare). Pure protein fractions were pooled together and concentrated to ~11 mg/ml.

CLK3 (~11 mg/ml) was co-crystallized with RN129 at 4°C using the sitting-drop vapor diffusion method by mixing protein and well solutions in 2:1, 1:1, and 1:2 ratios (drop size of 200 nl). The compound was incubated before crystallization with the protein for ~ 30 min on ice (final concentration of ~500 μM). The reservoir solution contained 15% (polyethylene glycol 6000) PEG 6k and 0.1 M Bicine buffer (pH 8). Ethylene glycol (25%) was used as cryoprotectant for crystals before flash freezing in liquid nitrogen. Diffraction data were collected at beamline X06SA (Swiss Light Source) at a wavelength of 1.0 Å at 100 K (https://doi.org/10.5281/zenodo.13934795). Data were processed using X-ray Detector Software DS (version 10 January 2022, http://xds.mpimf-heidelberg.mpg.de/, RRID:SCR_015652) (*49*) and scaled with aimless (version 0.8.2, www.ccp4.ac.uk/html/aimless.html, RRID:SCR_015747) (*50*). The PDB structure with the accession code 6YU1 (*51*) was used as an initial search molecular replacement (MR) model using the program MOLREP (version 11.0, www.ccp4.ac.uk/html/molrep.html) (*52*). The final model was built manually using Coot (version 0.8.9, www2.mrc-lmb.cam.ac.uk/personal/pemsley/coot/, RRID:SCR_014222) (*53*) and refined with REFMAC5 (www.ccp4.ac.uk/html/refmac5/description.html, RRID:SCR_014225) (*54*). Data collection and refinement statistics are summarized in table S5. Dictionary files for ligands were generated using the Grade Web Server (http://grade.globalphasing.org). (Protocol available at www.protocols.io/private/E313820E8AD611EFB8100A58A9FEAC02.)

### Cryo–electron microscopy

Purified LRRK2^RCKW^ was exchanged into 20 mM Hepes (pH 7.4), 150 mM NaCl, 0.5 mM TCEP, 5% glycerol, 2.5 mM MgCl_2_, and 20 μM GDP. LRRK2^RCKW^ was incubated with E11-DARPin (RRID: Addgene_226784), purified as previously described (*37*) in a 1:1.2 molar ratio and RN277 (30 μM) for 10 min at room temperature and 15 min at 4°C. Complex was diluted to a final concentration of 4.5 μM in the same buffer before plunge freezing. LRRK2^RCKW^:RN277:E11 DARPin complex (3 μl) was applied to a glow-discharged UltraAuFoil Holey Gold 200 mesh R2/2 grid (Quantifoil, Q250AR2A) and incubated in a FEI Vitrobot IV chamber at 4°C and 95% humidity for 20 s. The excess liquid was blotted for 4 s using filter paper 595 at blot force 5 and vitrified by plunging into liquid ethane cooled down to liquid nitrogen temperature. (Protocol available at dx.doi.org/10.17504/protocols.io.yxmvm35n9l3p/v1.)

Cryo-EM data for LRRK2^RCKW^:RN277:E11 DARPin were collected on a Titan Krios G3 (Thermo Fisher Scientific) operated at 300 keV, equipped with a Falcon 4 direct electron detector (Thermo Fisher Scientific) and a Gatan BioContinuum energy filter. Images were collected at defocus values varying between −1.0 and −2.0 μm at a nominal magnification of ×130,000 in energy filtered transmission electron microscope (EF-TEM) mode (0.935-Å calibrated pixel size) using a 20-eV slit width in the energy filter and a cumulative electron exposure of ~55 electrons/Å^2^. Data were collected automatically using EPU software (Thermo Fisher Scientific, www.thermofisher.com/us/en/home/electron-microscopy/products/software-em-3d-vis/epu-software.html). (Protocol available at dx.doi.org/10.17504/protocols.io.14egn3m5ql5d/v1.)

A total of 13,972 movies were collected for LRRK2^RCKW^:RN277:E11 DARPin and preprocessed using Patch MotionCor and Path CTF estimation to align and estimate contrast transfer function (CTF), respectively, both available in CryosPARC (*55*) (version 4.3, https://cryosparc.com/, RRID:SCR_016501). Micrographs with a CTF fit worse than 3.5 Å were excluded for further processing. Particles were picked using a Topaz (*56*) model (version 0.2.5, https://cb.csail.mit.edu/cb/topaz/) previously trained for the dataset. Several rounds of reference-free two-dimensional (2D) classification yielded a stack of 412,439 particles, which were further extracted with a 400-pixel box using CryoSPARC (fig. S1) (*55*). Subsequently, an ab initio and heterogeneous refinement jobs were run to remove bad particles. Particles from two output maps were used as an input for NU-Refinement (C1 symmetry). After 3D variability and local refinement, we obtained maps at 3.35 and 3.65 Å, respectively. In all maps, Fourier shell correlation (FSC) and local resolution estimations were performed using the routines implemented in CryoSPARC (*55*).

LRRK2^RCKW^ model was built using the highest-resolution local maps obtained for the complex. Available PDB 6VP7 was used as a starting point. Protein model was split into domains, docked into the corresponding cryo-EM maps using UCSF ChimeraX (*57*) (version 1.7, www.cgl.ucsf.edu/chimerax/, RRID:SCR_015872) and merged. For DARPin E11, we generated an initial model using ColabFold (*58*) (https://github.com/sokrypton/ColabFold, RRID:SCR_025453). To obtain an RN277 model, electronic Ligand Building and Optimization Workbench software available in Phenix (*59*) (version 1.21, www.phenix-online.org/, RRID:SCR_014224) was run using Simplified Molecular Input Line Entry System notation of molecule as an input. RN277 inhibitor model was fitted in the map density and incorporated in the model. A combination of manual inspection of amino acids in Coot (*53*, *60*) (version 0.8.9, www2.mrc-lmb.cam.ac.uk/personal/pemsley/coot/, RRID:SCR_014222) and refinement of model into their maps in Phenix (*59*) was used to generate the final model. Data collection and refinement statistics are summarized in table S3. All structure-related figures were prepared using ChimeraX (*57*). (Protocol available at dx.doi.org/10.17504/protocols.io.81wgbx5m1lpk/v1.)

### Kinome-wide selectivity profile

Compound RN341 was tested at two concentrations of 1 and 10 μM against a panel of 350 wild-type kinases in the ^33^PanQinase assay. The assay was performed by Reaction Biology (www.reactionbiology.com/services/kinase-assays/kinase-panel-screening/).

### nanoBRET off-target validation

The assay was performed as described previously (*61*). In brief, full-length kinases as listed in table S6 were obtained as plasmids cloned in frame with terminal NanoLuc-fusion (gift from Promega; table S6). Plasmids were transfected into human embryonic kidney 293T cells [American Type Culture Collection (ATCC), CRL-3216; RRID: CVCL_0063] using FuGENE 4 K (Promega, E5911), and proteins were allowed to express for 20 hours. Serially diluted inhibitor and corresponding tracer as listed in table S6 at the Tracer KD concentration taken from TracerDB (tracerdb.org) (*62*) were pipetted into white 384-well plates (Greiner, 781207) using an ECHO acoustic dispenser (Labcyte). The corresponding protein-transfected cells were added and reseeded at a density of 2 × 10^5^ cells/ml after trypsinization and resuspending in Opti-MEM without phenol red (Life Technologies). The system was allowed to equilibrate for 3 hours at 37°C/5% CO_2_ before bioluminescence resonance energy transfer (BRET) measurements. To measure BRET, NanoBRET NanoGlo Substrate + extracellular NanoLuc Inhibitor (Promega, N2540) was added as per the manufacturer’s protocol, and filtered luminescence was measured on a PHERAstar plate reader (BMG Labtech) equipped with a luminescence filter pair [450-nm BP filter (donor) and 610-nm LP filter (acceptor)]. Competitive displacement data were then graphed using GraphPad Prism 9 software (www.graphpad.com/, RRID:SCR_002798) using a normalized three-parameter curve fit with the following equation: *Y* = 100/[1 + 10(*X* − log *IC*_*50*_)]. (For step-by-step protocol, see www.eubopen.org/sites/www.eubopen.org/files/attachments/2021/NanoBRET-IC50_v1_0.pdf.)

### MS-based activity assay

To determine IC_50_ values for selected inhibitors, activity assay was performed using LRRK2^RCKW^ and Rab8A substrate (table S4). LRRK2^RCKW^ (20 nM) was incubated with 5 μM substrate and a concentration series of the respective inhibitor in 20 mM Hepes (pH 7.4), 100 mM NaCl, 0.5 mM TCEP, 0.5% glycerol, 20 μM GDP, and 2.5 mM MgCl_2_. The reaction was started by adding 1 mM ATP and incubated at room temperature for 4 hours. The phosphorylation reaction was stopped by addition of MS buffer (0.1% formic acid in water). Reaction mix (5 μl) was injected into an Agilent 6230 electrospray ionization time-of-flight mass spectrometer coupled to a 1260 Infinity liquid chromatography unit (flow rate at 0.4 ml/min using a solvent gradient of water to acetonitrile with 0.1% formic acid). Data were acquired using the MassHunter LC/MS Data Acquisition software and analyzed using the BioConfirm vB.08.00 tool (both Agilent Technology, www.agilent.com/en/product/software-informatics/mass-spectrometry-software, RRID: SCR_016657).

Peak intensities of unphosphorylated and phosphorylated Rab8A were quantified, and the relative kinase activity was calculated as a ratio. To determine the IC_50_, a nonlinear regression with variable slope was fitted to the datapoints with GraphPad Prism (www.graphpad.com/, RRID:SCR_002798). (For step-by-step protocol, see dx.doi.org/10.17504/protocols.io.6qpvr385ovmk/v2.)

### Cell treatments and Western blot analysis

For Western blot analysis of LRRK2 kinase activity in cells, 293T cells (ATCC, CRL-3216; RRID: CVCL_0063) were transfected with 1000 ng of wild-type full-length LRRK2 (pcDNA5-LRRK2, RRID: Addgene_229019) and 500 ng of GFP-Rab8A (RRID: Addgene_49543) using polyethylenimine (Polysciences). For analysis of inhibitor activity against wild-type and G2019S mutant LRRK2, 293T cells were transfected with 500 ng of GFP-Rab8a and either 1000 ng of GFP-11 tagged wild-type LRRK2 (pcDNA5-FRT-TO-GFP11-LRRK2, RRID: Addgene_231174) or GFP-11 tagged G2019S LRRK2 (pcDNA5-FRT-TO-GFP11-LRRK2-G2019S, RRID: Addgene_231175). For Western blot analysis of inhibitor activity against LRRK1, 293T cells were transfected with 1000 ng of FLAG-LRRK1 construct (RRID: Addgene_232062) and 500 ng of GFP-Rab7 (RRID: Addgene_12605) (table S4).

For all in cell kinase assays, after 48 hours, transfected cells were treated with either DMSO (control) or the indicated inhibitors for 4 hours at 37°C and 5% CO_2_. Following treatments, cells were washed three times with ice cold PBS. Cells were then lysed in ice cold radioimmunoprecipitation assay (RIPA) lysis buffer [50 mM tris (pH 8.0), 150 mM NaCl, 1% Triton X-100, 0.1% SDS, 0.5% sodium deoxycholate, and 1 mM DTT] in the presence of complete mini EASYpack protease inhibitor cocktail and the PhosSTOP EASYpack phosphatase inhibitor cocktail (Roche). Resuspended and lysed cells were clarified by centrifugation at 13,000*g* for 15 min at 4°C. NuPAGE LDS Sample Buffer (1×) and NuPAGE Sample Reducing Agent (1×; Invitrogen) were added to the clarified supernatants, and the samples were boiled at 95°C for 5 min and spun down at 13,000*g* for 10 min. For Western blotting, 15 μl of sample was loaded into a NuPAGE 4 to 12% gradient bis-tris gel (Invitrogen) and run at 120 V for 75 min before transfer to polyvinylidene difluoride transfer membrane (0.45-μm pore size, Millipore) for 240 min at 80 V at 4°C. The membrane was blocked in 5% milk in 1× Tris-buffered saline (TBS) [20 mM tris (pH 8.0) and 150 mM NaCl] for 1 hour shaking at room temperature. Primary antibodies [monoclonal rabbit anti-LRRK2 (Abcam, ab133474; RRID: AB_2713963), polyclonal rabbit anti-LRRK1 (Abcam, ab228666), monoclonal rabbit anti-LRRK2 phospho-S935 (Abcam, ab133450; RRID: AB_2732035), monoclonal rabbit anti-Rab8a phospho-T72 (Abcam, ab230260; RRID: AB_2814988), monoclonal rabbit anti-Rab7 phospho-S72 (Abcam, ab302494; RRID: AB_2933985), monoclonal rabbit anti–glyceraldehyde-3-phosphate dehydrogenase (GAPDH; Cell Signalling Technology, 2118; RRID: AB_561053), and monoclonal mouse anti-GFP (Santa Cruz Biotechnology, sc-9996; RRID: AB_627695)] were diluted in 5% milk in TBS and incubated with membrane for 16 hours at 4°C. Following incubation, membranes were washed three times with 1× TBS-T [20 mM tris (pH 8.0), 150 mM NaCl, and 0.1% Tween 20] before incubation with IRDye 680RD goat anti-rabbit IgG (LI-COR, 926-68071; RRID: AB_10956166) and IRDye 800CW goat anti-mouse IgG (LI-COR, 926-32210; RRID: AB_621842) secondary antibodies in 5% milk in 1× TBS for 1 hour at room temperature. Membranes were then washed three times in 1× TBS-T before imaging on Li-COR Odyssey CLx controlled by Imaging Studio software (www.licorbio.com/image-studio, RRID:SCR_015795). Image processing and quantification were performed in ImageJ version 2.14.0 (National Institutes of Health; https://imagej.net/, RRID:SCR_003070). The signal intensity of each band was assessed using the Analyze>Gels tool. The band intensity for phosphorylated LRRK2 was measured and compared to total LRRK2 levels (run under identical conditions on a separate gel). Band intensity for phosphorylated Rab7 or Rab8a was measured and compared against total GFP-Rab7 or GFP-Rab8a (using anti-GFP antibody) and LRRK1/LRRK2 levels as appropriate. Comparative statistical analysis of phosphorylation levels was analyzed using a one-way analysis of variance (ANOVA) and corrected using Tukey’s multiple comparisons test in GraphPad Prism 10 (www.graphpad.com/, RRID:SCR_002798). (For current step-by-step protocol for LRRK transfection and Western blotting, see dx.doi.org/10.17504/protocols.io.kxygx9bozg8j/v1.)

### TIRF microscopy

Imaging for single-molecule motility assays was performed with an inverted microscope (Nikon, Ti-E Eclipse) equipped with a 100× 1.49 numerical aperture oil immersion objective (Nikon, Plano Apo) and a MLC400B laser launch (Agilent), with 405-, 488-, 561-, and 640-nm laser lines. The excitation and emission paths were filtered using appropriate single band-pass filter cubes (Chroma). The emitted signals were detected with an electron multiplying charge-coupled device camera (Andor Technology, iXon Ultra 888), and the stage *xy* position was controlled by a ProScan linear motor stage controller (Prior). Illumination and image acquisition was controlled by NIS Elements Advanced Research software (Nikon; www.microscope.healthcare.nikon.com/products/software/nis-elements/nis-elements-advanced-research, RRID:SCR_014329).

### Single-molecule motility assays

Single-molecule total internal reflection fluorescence (TIRF) motility experiments were performed in flow chambers using the microscopy setup described above. Flow chambers were made by adhering 1^1^/_2_ thickness cover glass (Corning) to a SuperFrost Plus microscope slide (Electron Microscopy Sciences) with double sided tape. Before flow chamber assembly, cover glass was washed in 1 M HCl at 60°C for 1 hour and sonicated in 100% ethanol for 10 min to reduce nonspecific binding. Taxol-stabilized microtubules composed of ~10% biotin-tubulin (Cytoskeleton) for attachment to streptavidin-coated cover glass and ~10% Alexa Fluor 405 (Thermo Fisher Scientific)–tubulin for visualization were prepared, and motility assays were performed as previously described (*22*). In brief, flow chambers were functionalized by incubation with biotin-BSA (1 mg/ml; Sigma-Aldrich) for 3 min, followed by streptavidin (0.5 mg/ml) for a further 3 min. Taxol-stabilized microtubules were diluted 1:100 in motility assay buffer [30 mM Hepes (pH 7.4), 50 mM potassium acetate, 2 mM magnesium acetate, 1 mM EGTA, 10% glycerol, 1 mM DTT, and 20 μM Taxol] and incubated with the flow chamber for 3 min. Following incubation, the flow chambers with adhered microtubules were washed three times with LRRK2 buffer [20 mM Hepes (pH 7.4), 80 mM NaCl, 0.5 mM TCEP, 5% glycerol, 2.5 mM MgCl_2_, and 20 μM GDP] before incubation for 5 min with either LRRK2 buffer with DMSO or specified kinase inhibitors (0 nM LRRK2^RCKW^) or LRRK2 buffer containing LRRK2^RCKW^ and DMSO or kinase inhibitors as indicated. In all cases, DMSO or the indicated kinase inhibitors were incubated with LRRK2 buffer in the presence or absence of LRRK2^RCKW^ for 10 min before addition and incubation within the flow chambers. Flow chambers were then washed with motility assay buffer supplemented with casein (1 mg/ml) and K560-GFP added in the final imaging buffer [motility assay buffer supplemented with an oxygen scavenger system of glucose catalase (45 μg ml^−1^; Sigma-Aldrich) and glucose oxidase (1.15 mg ml^−1^; Sigma-Aldrich), 0.4% glucose, 71.5 mM β-mercaptoethanol, and 1 mM ATP]. The final concentration of K560-GFP in the flow chamber was 1.9 nM. K560-GFP was imaged every 500 ms for 2 min with 20% laser (488) power. (Protocol available at dx.doi.org/10.17504/protocols.io.5qpvok4dzl4o/v2.)

### TIRF motility data analysis

Data were blinded before analysis. Kymographs were generated from motility movies and quantified for percent motility using ImageJ V.2.14.0 as previously described (*22*, *63*). Processive, diffusive, and nonmotile events were manually annotated for calculation of the percentage of processive events per microtubule. Processive events were defined as runs that moved unidirectionally and did not exhibit directional changes. Diffusive events were those with at least one bidirectional change greater than 600 nm in each direction, and nonmotile events were defined as those that did not exhibit movement. Single-molecule movements with multiple behaviors were counted as multiple events. For percentage motility per microtubule measurements, processive events were divided by total events (nonmotile, diffuse, and processive) per kymograph.

For statistical analysis, brightness and contrast were adjusted in ImageJ for all motility movies and kymographs. Statistical analyses were performed in GraphPad Prism 10 (www.graphpad.com/, RRID:SCR_002798). Specific statistical analysis descriptors, *n* values, and *P* values can be found in the corresponding figure legends. All TIRF experiments were analyzed from three independent technical replicates.

## Supplementary Materials

### The PDF file includes:

Figs. S1 to S8 Tables S1 to S6 Legend for data S1 Synthesis Instructions NMR spectra HPLC/MS HRMS

## References

[1] A. W. Willis, E. Roberts, J. C. Beck, B. Fiske, W. Ross, R. Savica, S. K. Van Den Eeden, C. M. Tanner, C. Marras, Parkinson’s Foundation P4 Group, Incidence of Parkinson disease in North America. NPJ Parkinson Dis. 8, 170 (2022). 10.1038/s41531-022-00410-y PMC9755252 36522332

[2] GBD 2016 Neurology Collaborators, Global, regional, and national burden of neurological disorders, 1990-2016: A systematic analysis for the Global Burden of Disease Study 2016. Lancet Neurol. 18, 459–480 (2019). 30879893 10.1016/S1474-4422(18)30499-X PMC6459001

[3] T. Hatano, S. Kubo, S. Sato, N. Hattori, Pathogenesis of familial Parkinson’s disease: New insights based on monogenic forms of Parkinson’s disease. J. Neurochem. 111, 1075–1093 (2009). 19780902 10.1111/j.1471-4159.2009.06403.x

[4] C. Schiesling, N. Kieper, K. Seidel, R. Krüger, Review: Familial Parkinson’s disease – genetics, clinical phenotype and neuropathology in relation to the common sporadic form of the disease. Neuropathol. Appl. Neurobiol. 34, 255–271 (2008). 18447897 10.1111/j.1365-2990.2008.00952.x

[5] C. Paisán-Ruíz, S. Jain, E. W. Evans, W. P. Gilks, J. Simón, M. Van Der Brug, A. L. De Munain, S. Aparicio, A. M. Gil, N. Khan, J. Johnson, J. R. Martinez, D. Nicholl, I. M. Carrera, A. S. Peňa, R. De Silva, A. Lees, J. F. Eartí-Massó, J. Pérez-Tur, N. W. Wood, A. B. Singleton, Cloning of the gene containing mutations that cause PARK8-linked Parkinson’s disease. Neuron 44, 595–600 (2004). 15541308 10.1016/j.neuron.2004.10.023

[6] A. Zimprich, S. Biskup, P. Leitner, P. Lichtner, M. Farrer, S. Lincoln, J. Kachergus, M. Hulihan, R. J. Uitti, D. B. Calne, A. J. Stoessl, R. F. Pfeiffer, N. Patenge, I. C. Carbajal, P. Vieregge, F. Asmus, B. Müller-Myhsok, D. W. Dickson, T. Meitinger, T. M. Strom, Z. K. Wszolek, T. Gasser, Mutations in LRRK2 cause autosomal-dominant parkinsonism with pleomorphic pathology. Neuron 44, 601–607 (2004). 15541309 10.1016/j.neuron.2004.11.005

[7] W. Satake, Y. Nakabayashi, I. Mizuta, Y. Hirota, C. Ito, M. Kubo, T. Kawaguchi, T. Tsunoda, M. Watanabe, A. Takeda, H. Tomiyama, K. Nakashima, K. Hasegawa, F. Obata, T. Yoshikawa, H. Kawakami, S. Sakoda, M. Yamamoto, N. Hattori, M. Murata, Y. Nakamura, T. Toda, Genome-wide association study identifies common variants at four loci as genetic risk factors for Parkinson’s disease. Nat. Genet. 41, 1303–1307 (2009). 19915576 10.1038/ng.485

[8] J. Simón-Sánchez, C. Schulte, J. M. Bras, M. Sharma, J. R. Gibbs, D. Berg, C. Paisan-Ruiz, P. Lichtner, S. W. Scholz, D. G. Hernandez, R. Krüger, M. Federoff, C. Klein, A. Goate, J. Perlmutter, M. Bonin, M. A. Nalls, T. Illig, C. Gieger, H. Houlden, M. Steffens, M. S. Okun, B. A. Racette, M. R. Cookson, K. D. Foote, H. H. Fernandez, B. J. Traynor, S. Schreiber, S. Arepalli, R. Zonozi, K. Gwinn, M. Van Der Brug, G. Lopez, S. J. Chanock, A. Schatzkin, Y. Park, A. Hollenbeck, J. Gao, X. Huang, N. W. Wood, D. Lorenz, G. Deuschl, H. Chen, O. Riess, J. A. Hardy, A. B. Singleton, T. Gasser, Genome-wide association study reveals genetic risk underlying Parkinson’s disease. Nat. Genet. 41, 1308–1312 (2009). 19915575 10.1038/ng.487 PMC2787725

[9] R. Di Maio, E. K. Hoffman, E. M. Rocha, M. T. Keeney, L. H. Sanders, B. R. De Miranda, A. Zharikov, A. Van Laar, A. F. Stepan, T. A. Lanz, J. K. Kofler, E. A. Burton, D. R. Alessi, T. G. Hastings, J. T. Greenamyre, LRRK2 activation in idiopathic Parkinson’s disease. Sci. Transl. Med. 10, eaar5429 (2018). 30045977 10.1126/scitranslmed.aar5429 PMC6344941

[10] A. Di Fonzo, C. F. Rohé, J. Ferreira, H. F. Chien, L. Vacca, F. Stocchi, L. Guedes, E. Fabrizio, M. Manfredi, N. Vanacore, S. Goldwurm, G. Breedveld, C. Sampaio, G. Meco, E. Barbosa, B. A. Oostra, V. Bonifati, A frequent LRRK2 gene mutation associated with autosomal dominant Parkinson’s disease. Lancet 365, 412–415 (2005). 15680456 10.1016/S0140-6736(05)17829-5

[11] W. C. Nichols, N. Pankratz, D. Hernandez, C. Paisán-Ruíz, S. Jain, C. A. Halter, V. E. Michaels, T. Reed, A. Rudolph, C. W. Shults, A. Singleton, T. Foroud, Genetic screening for a single common LRRK2 mutation in familial Parkinson’s disease. Lancet 365, 410–412 (2005). 15680455 10.1016/S0140-6736(05)17828-3

[12] W. P. Gilks, P. M. Abou-Sleiman, S. Gandhi, S. Jain, A. Singleton, A. J. Lees, K. Shaw, K. P. Bhatia, V. Bonifati, N. P. Quinn, J. Lynch, D. G. Healy, J. L. Holton, T. Revesz, N. W. Wood, A common LRRK2 mutation in idiopathic Parkinson’s disease. Lancet 365, 415–416 (2005). 15680457 10.1016/S0140-6736(05)17830-1

[13] A. B. West, D. J. Moore, S. Biskup, A. Bugayenko, W. W. Smith, C. A. Ross, V. L. Dawson, T. M. Dawson, Parkinson’s disease-associated mutations in leucine-rich repeat kinase 2 augment kinase activity. Proc. Natl. Acad. Sci. U.S.A. 102, 16842–16847 (2005). 16269541 10.1073/pnas.0507360102 PMC1283829

[14] C. C. Ayala-Aguilera, T. Valero, Á. Lorente-Macías, D. J. Baillache, S. Croke, A. Unciti-Broceta, Small molecule kinase inhibitor drugs (1995–2021): Medical indication, pharmacology, and synthesis. J. Med. Chem. 65, 1047–1131 (2022). 34624192 10.1021/acs.jmedchem.1c00963

[15] X. Deng, N. Dzamko, A. Prescott, P. Davies, Q. Liu, Q. Yang, J.-D. Lee, M. P. Patricelli, T. K. Nomanbhoy, D. R. Alessi, N. S. Gray, Characterization of a selective inhibitor of the Parkinson’s disease kinase LRRK2. Nat. Chem. Biol. 7, 203–205 (2011). 21378983 10.1038/nchembio.538 PMC3287420

[16] N. Ramsden, J. Perrin, Z. Ren, B. D. Lee, N. Zinn, V. L. Dawson, D. Tam, M. Bova, M. Lang, G. Drewes, M. Bantscheff, F. Bard, T. M. Dawson, C. Hopf, Chemoproteomics-based design of potent LRRK2-selective lead compounds that attenuate Parkinson’s disease-related toxicity in human neurons. ACS Chem. Biol. 6, 1021–1028 (2011). 21812418 10.1021/cb2002413 PMC3688284

[17] A. A. Estrada, X. Liu, C. Baker-Glenn, A. Beresford, D. J. Burdick, M. Chambers, B. K. Chan, H. Chen, X. Ding, A. G. DiPasquale, S. L. Dominguez, J. Dotson, J. Drummond, M. Flagella, S. Flynn, R. Fuji, A. Gill, J. Gunzner-Toste, S. F. Harris, T. P. Heffron, T. Kleinheinz, D. W. Lee, C. E. Le Pichon, J. P. Lyssikatos, A. D. Medhurst, J. G. Moffat, S. Mukund, K. Nash, K. Scearce-Levie, Z. Sheng, D. G. Shore, T. Tran, N. Trivedi, S. Wang, S. Zhang, X. Zhang, G. Zhao, H. Zhu, Z. K. Sweeney, Discovery of highly potent, selective, and brain-penetrable leucine-rich repeat kinase 2 (LRRK2) small molecule inhibitors. J. Med. Chem. 55, 9416–9433 (2012). 22985112 10.1021/jm301020q

[18] J. L. Henderson, B. L. Kormos, M. M. Hayward, K. J. Coffman, J. Jasti, R. G. Kurumbail, T. T. Wager, P. R. Verhoest, G. S. Noell, Y. Chen, E. Needle, Z. Berger, S. J. Steyn, C. Houle, W. D. Hirst, P. Galatsis, Discovery and preclinical profiling of 3-[4-(Morpholin-4-yl)-7*H*-pyrrolo[2,3-*d*]pyrimidin-5-yl]benzonitrile (PF-06447475), a highly potent, selective, brain penetrant, and in vivo active LRRK2 kinase inhibitor. J. Med. Chem. 58, 419–432 (2015). 25353650 10.1021/jm5014055

[19] M. J. Fell, C. Mirescu, K. Basu, B. Cheewatrakoolpong, D. E. DeMong, J. M. Ellis, L. A. Hyde, Y. Lin, C. G. Markgraf, H. Mei, M. Miller, F. M. Poulet, J. D. Scott, M. D. Smith, Z. Yin, X. Zhou, E. M. Parker, M. E. Kennedy, J. A. Morrow, MLi-2, a potent, selective, and centrally active compound for exploring the therapeutic potential and safety of LRRK2 kinase inhibition. J. Pharmacol. Exp. Ther. 355, 397–409 (2015). 26407721 10.1124/jpet.115.227587

[20] A. Tasegian, F. Singh, I. G. Ganley, A. D. Reith, D. R. Alessi, Impact of Type II LRRK2 inhibitors on signalling and mitophagy. Biochem. J. 478, 3555–3573 (2021). 34515301 10.1042/BCJ20210375 PMC8589421

[21] M. S. Murillo, A. V. Suarez, V. Dederer, D. Chatterjee, J. A. Louro, S. Knapp, S. Mathea, A. E. Leschziner, Inhibition of Parkinson’s disease-related LRRK2 by type I and type II kinase inhibitors: Activity and structures. Sci. Adv. 9, eadk6191 (2023). 38039358 10.1126/sciadv.adk6191 PMC10691770

[22] C. K. Deniston, J. Salogiannis, S. Mathea, D. M. Snead, I. Lahiri, M. Matyszewski, O. Donosa, R. Watanabe, J. Böhning, A. K. Shiau, S. Knapp, E. Villa, S. L. Reck-Peterson, A. E. Leschziner, Structure of LRRK2 in Parkinson’s disease and model for microtubule interaction. Nature 588, 344–349 (2020). 32814344 10.1038/s41586-020-2673-2 PMC7726071

[23] Z. Zhao, H. Wu, L. Wang, Y. Liu, S. Knapp, Q. Liu, N. S. Gray, Exploration of type II binding mode: A privileged approach for kinase inhibitor focused drug discovery? ACS Chem. Biol. 9, 1230–1241 (2014). 24730530 10.1021/cb500129t PMC4068218

[24] R. S. K. Vijayan, P. He, V. Modi, K. C. Duong-Ly, H. Ma, J. R. Peterson, R. L. Dunbrack, R. M. Levy, Conformational analysis of the DFG-out kinase motif and biochemical profiling of structurally validated type II inhibitors. J. Med. Chem. 58, 466–479 (2015). 25478866 10.1021/jm501603h PMC4326797

[25] H. Zhu, P. Hixson, W. Ma, J. Sun, Pharmacology of LRRK2 with type I and II kinase inhibitors revealed by cryo-EM. Cell Discov. 10, 10 (2024). 38263358 10.1038/s41421-023-00639-8 PMC10805800

[26] R. Watanabe, R. Buschauer, J. Böhning, M. Audagnotto, K. Lasker, T.-W. Lu, D. Boassa, S. Taylor, E. Villa, The in situ structure of Parkinson’s disease-linked LRRK2. Cell 182, 1508–1518.e16 (2020). 32783917 10.1016/j.cell.2020.08.004 PMC7869717

[27] S. H. Schmidt, J.-H. Weng, P. C. Aoto, D. Boassa, S. Mathea, S. Silletti, J. Hu, M. Wallbott, E. A. Komives, S. Knapp, F. W. Herberg, S. S. Taylor, Conformation and dynamics of the kinase domain drive subcellular location and activation of LRRK2. Proc. Natl. Acad. Sci. U.S.A. 118, e2100844118 (2021). 34088839 10.1073/pnas.2100844118 PMC8201809

[28] D. M. Snead, M. Matyszewski, A. M. Dickey, Y. X. Lin, A. E. Leschziner, S. L. Reck-Peterson, Structural basis for Parkinson’s disease-linked LRRK2’s binding to microtubules. Nat. Struct. Mol. Biol. 29, 1196–1207 (2022). 36510024 10.1038/s41594-022-00863-y PMC9758056

[29] Z. Liu, N. Bryant, R. Kumaran, A. Beilina, A. Abeliovich, M. R. Cookson, A. B. West, LRRK2 phosphorylates membrane-bound Rabs and is activated by GTP-bound Rab7L1 to promote recruitment to the trans-Golgi network. Hum. Mol. Genet. 27, 385–395 (2018). 29177506 10.1093/hmg/ddx410 PMC5886198

[30] Z. Berger, K. A. Smith, M. J. LaVoie, Membrane localization of LRRK2 is associated with increased formation of the highly active LRRK2 dimer and changes in its phosphorylation. Biochemistry 49, 5511–5523 (2010). 20515039 10.1021/bi100157u PMC2987719

[31] L. Bonet-Ponce, A. Beilina, C. D. Williamson, E. Lindberg, J. H. Kluss, S. Saez-Atienzar, N. Landeck, R. Kumaran, A. Mamais, C. K. E. Bleck, Y. Li, M. R. Cookson, LRRK2 mediates tubulation and vesicle sorting from lysosomes. Sci. Adv. 6, eabb2454 (2020). 33177079 10.1126/sciadv.abb2454 PMC7673727

[32] C. J. Gloeckner, N. Kinkl, A. Schumacher, R. J. Braun, E. O’Neill, T. Meitinger, W. Kolch, H. Prokisch, M. Ueffing, The Parkinson disease causing LRRK2 mutation I2020T is associated with increased kinase activity. Hum. Mol. Genet. 15, 223–232 (2006). 16321986 10.1093/hmg/ddi439

[33] L. R. Kett, D. Boassa, C. C.-Y. Ho, H. J. Rideout, J. Hu, M. Terada, M. Ellisman, W. T. Dauer, LRRK2 Parkinson disease mutations enhance its microtubule association. Hum. Mol. Genet. 21, 890–899 (2012). 22080837 10.1093/hmg/ddr526 PMC3263991

[34] A. Tasegian, F. Singh, I. G. Ganley, A. D. Reith, D. R. Alessi, Impact of type II LRRK2 inhibitors on signaling and mitophagy. Biochem. J. 478, 3555–3573 (2021). 34515301 10.1042/BCJ20210375 PMC8589421

[35] R. N. Fuji, M. Flagella, M. Baca, M. A. S. Baptista, J. Brodbeck, B. K. Chan, B. K. Fiske, L. Honigberg, A. M. Jubb, P. Katavolos, D. W. Lee, S.-C. Lewin-Koh, T. Lin, X. Liu, S. Liu, J. P. Lyssikatos, J. O’Mahony, M. Reichelt, M. Roose-Girma, Z. Sheng, T. Sherer, A. Smith, M. Solon, Z. K. Sweeney, J. Tarrant, A. Urkowitz, S. Warming, M. Yaylaoglu, S. Zhang, H. Zhu, A. A. Estrada, R. J. Watts, Effect of selective LRRK2 kinase inhibition on nonhuman primate lung. Sci. Transl. Med. 7, 273ra15 (2015). 10.1126/scitranslmed.aaa3634 25653221

[36] M. A. S. Baptista, K. D. Dave, M. A. Frasier, T. B. Sherer, M. Greeley, M. J. Beck, J. S. Varsho, G. A. Parker, C. Moore, M. J. Churchill, C. K. Meshul, B. K. Fiske, Loss of leucine-rich repeat kinase 2 (LRRK2) in rats leads to progressive abnormal phenotypes in peripheral organs. PLOS ONE 8, e80705 (2013). 24244710 10.1371/journal.pone.0080705 PMC3828242

[37] V. Dederer, M. Sanz Murillo, E. P. Karasmanis, K. S. Hatch, D. Chatterjee, F. Preuss, K. R. Abdul Azeez, L. V. Nguyen, C. Galicia, B. Dreier, A. Plückthun, W. Versees, S. Mathea, A. E. Leschziner, S. L. Reck-Peterson, S. Knapp, A designed ankyrin-repeat protein that targets Parkinson’s disease-associated LRRK2. J. Biol. Chem. 300, 107469 (2024). 38876305 10.1016/j.jbc.2024.107469 PMC11284679

[38] M. B. Robers, R. Friedman-Ohana, K. V. M. Huber, L. Kilpatrick, J. D. Vasta, B.-T. Berger, C. Chaudhry, S. Hill, S. Müller, S. Knapp, K. V. Wood, Quantifying target occupancy of small molecules within living cells. Annu. Rev. Biochem. 89, 557–581 (2020). 32208767 10.1146/annurev-biochem-011420-092302

[39] J. M. Reimer, A. M. Dickey, Y. X. Lin, R. G. Abrisch, S. Mathea, D. Chatterjee, E. J. Fay, S. Knapp, M. D. Daugherty, S. L. Reck-Peterson, A. E. Leschziner, Structure of LRRK1 and mechanisms of autoinhibition and activation. Nat. Struct. Mol. Biol. 30, 1735–1745 (2023). 37857821 10.1038/s41594-023-01109-1 PMC10643122

[40] W. R. Xing, H. Goodluck, C. Zeng, S. Mohan, Role and mechanism of action of leucine-rich repeat kinase 1 in bone. Bone Res. 5, 17003 (2017). 28326224 10.1038/boneres.2017.3 PMC5348726

[41] A. Iida, W. Xing, M. K. F. Docx, T. Nakashima, Z. Wang, M. Kimizuka, W. Van Hul, D. Rating, J. Spranger, H. Ohashi, N. Miyake, N. Matsumoto, S. Mohan, G. Nishimura, G. Mortier, S. Ikegawa, Identification of biallelic *LRRK1* mutations in osteosclerotic metaphyseal dysplasia and evidence for locus heterogeneity. J. Med. Genet. 53, 568–574 (2016). 27055475 10.1136/jmedgenet-2016-103756 PMC5769692

[42] E. F. S. Van Velsen, S. Demirdas, D. Hanff, M. C. Zillikens, osteosclerotic metaphyseal dysplasia due to a likely pathogenic LRRK1 variant as a cause of recurrent long bone fractures. JBMR Plus 7, e10755 (2023). 37614307 10.1002/jbm4.10755 PMC10443074

[43] M. Steger, F. Tonelli, G. Ito, P. Davies, M. Trost, M. Vetter, S. Wachter, E. Lorentzen, G. Duddy, S. Wilson, M. A. Baptista, B. K. Fiske, M. J. Fell, J. A. Morrow, A. D. Reith, D. R. Alessi, M. Mann, Phosphoproteomics reveals that Parkinson’s disease kinase LRRK2 regulates a subset of Rab GTPases. eLife 5, e12813 (2016). 26824392 10.7554/eLife.12813 PMC4769169

[44] S. Müller, A. Chaikuad, N. S. Gray, S. Knapp, The ins and outs of selective kinase inhibitor development. Nat. Chem. Biol. 11, 818–821 (2015). 26485069 10.1038/nchembio.1938

[45] S. Shyam Sunder, U. C. Sharma, S. Pokharel, Adverse effects of tyrosine kinase inhibitors in cancer therapy: Pathophysiology, mechanisms and clinical management. Signal Transduct. Target. Ther. 8, 262 (2023). 37414756 10.1038/s41392-023-01469-6 PMC10326056

[46] K. J. Rangan, S. L. Reck-Peterson, RNA recoding in cephalopods tailors microtubule motor protein function. Cell 186, 2531–2543.e11 (2023). 37295401 10.1016/j.cell.2023.04.032 PMC10467349

[47] F. H. Niesen, H. Berglund, M. Vedadi, The use of differential scanning fluorimetry to detect ligand interactions that promote protein stability. Nat. Protoc. 2, 2212–2221 (2007). 17853878 10.1038/nprot.2007.321

[48] J. A. Amrhein, L. M. Berger, A. Tjaden, A. Krämer, L. Elson, T. Tolvanen, D. Martinez-Molina, A. Kaiser, M. Schubert-Zsilavecz, S. Müller, S. Knapp, T. Hanke, Discovery of 3-amino-1h-pyrazole-based kinase inhibitors to illuminate the understudied PCTAIRE family. Int. J. Mol. Sci. 23, 14834 (2022). 36499165 10.3390/ijms232314834 PMC9736855

[49] W. Kabsch, XDS. Acta Crystallogr. D Biol. Crystallogr. 66, 125–132 (2010). 20124692 10.1107/S0907444909047337 PMC2815665

[50] P. R. Evans, G. N. Murshudov, How good are my data and what is the resolution? Acta Crystallogr. D Biol. Crystallogr. 69, 1204–1214 (2013). 23793146 10.1107/S0907444913000061 PMC3689523

[51] M. Schröder, A. N. Bullock, O. Fedorov, F. Bracher, A. Chaikuad, S. Knapp, DFG-1 residue controls inhibitor binding mode and affinity, providing a basis for rational design of kinase inhibitor selectivity. J. Med. Chem. 63, 10224–10234 (2020). 32787076 10.1021/acs.jmedchem.0c00898

[52] A. A. Lebedev, A. A. Vagin, G. N. Murshudov, Model preparation in MOLREP and examples of model improvement using X-ray data. Acta Crystallogr. D Biol. Crystallogr. 64, 33–39 (2007). 18094465 10.1107/S0907444907049839 PMC2394799

[53] P. Emsley, K. Cowtan, Coot: Model-building tools for molecular graphics. Acta Crystallogr. D Biol. Crystallogr. 60, 2126–2132 (2004). 15572765 10.1107/S0907444904019158

[54] A. A. Vagin, R. A. Steiner, A. A. Lebedev, L. Potterton, S. McNicholas, F. Long, G. N. Murshudov, REFMAC5 dictionary: Organization of prior chemical knowledge and guidelines for its use. Acta Crystallogr. D Biol. Crystallogr. 60, 2184–2195 (2004). 15572771 10.1107/S0907444904023510

[55] A. Punjani, J. L. Rubinstein, D. J. Fleet, M. A. Brubaker, cryoSPARC: Algorithms for rapid unsupervised cryo-EM structure determination. Nat. Methods 14, 290–296 (2017). 28165473 10.1038/nmeth.4169

[56] T. Bepler, A. Morin, M. Rapp, J. Brasch, L. Shapiro, A. J. Noble, B. Berger, TOPAZ: A positive-unlabeled convolutional neural network CryoEM particle picker that can pick any size and shape particle. Microsc. Microanal. 25, 986–987 (2019).

[57] E. F. Pettersen, T. D. Goddard, C. C. Huang, E. C. Meng, G. S. Couch, T. I. Croll, J. H. Morris, T. E. Ferrin, UCSF ChimeraX: Structure visualization for researchers, educators, and developers. Protein Sci. 30, 70–82 (2020). 32881101 10.1002/pro.3943 PMC7737788

[58] M. Mirdita, K. Schütze, Y. Moriwaki, L. Heo, S. Ovchinnikov, M. Steinegger, ColabFold: Making protein folding accessible to all. Nat. Methods 19, 679–682 (2022). 35637307 10.1038/s41592-022-01488-1 PMC9184281

[59] D. Liebschner, P. V. Afonine, M. L. Baker, G. Bunkóczi, V. B. Chen, T. I. Croll, B. Hintze, L.-W. Hung, S. Jain, A. J. McCoy, N. W. Moriarty, R. D. Oeffner, B. K. Poon, M. G. Prisant, R. J. Read, J. S. Richardson, D. C. Richardson, M. D. Sammito, O. V. Sobolev, P. D. Adams, Macromolecular structure determination using Xrays, neutrons and electrons: Recent developments in Phenix. Acta Crystallogr. D Struct. Biol. 75, 861–877 (2019). 31588918 10.1107/S2059798319011471 PMC6778852

[60] P. Emsley, B. Lohkamp, W. G. Scott, K. Cowtan, Features and development of Coot. Acta Crystallogr. D Biol. Crystallogr. 66, 486–501 (2010). 20383002 10.1107/S0907444910007493 PMC2852313

[61] M. P. Schwalm, K. Saxena, S. Müller, S. Knapp, Luciferase- and HaloTagbased reporter assays to measure small-molecule-induced degradation pathway in living cells. Nat. Protoc. 19, 2317–2357 (2024). 38637703 10.1038/s41596-024-00979-z

[62] M. Schwalm, J. Dopfer, J. Vasta, S. Muller, S. Knapp, M. Robers, tracerDB: A crowdsourced fluorescent tracer database for target engagement analysis. Nat. Commun. 15, 5646 (2024). 38969708 10.1038/s41467-024-49896-5 PMC11226670

[63] A. J. Roberts, B. S. Goodman, S. L. Reck-Peterson, Reconstitution of dynein transport to the microtubule plus end by kinesin. eLife 3, e02641 (2014). 24916158 10.7554/eLife.02641 PMC4046564

